# High Meiofaunal and Nematodes Diversity around Mesophotic Coral Oases in the Mediterranean Sea

**DOI:** 10.1371/journal.pone.0066553

**Published:** 2013-06-18

**Authors:** Silvia Bianchelli, Antonio Pusceddu, Simone Canese, Silvio Greco, Roberto Danovaro

**Affiliations:** 1 Dipartimento di Scienze della Vita e dell'Ambiente, Università Politecnica delle Marche, Ancona, Italy; 2 Istituto Superiore per la Ricerca Ambientale, ISPRA, Roma, Italy; Bangor University, United Kingdom

## Abstract

Although the mesophotic zone of the Mediterranean Sea has been poorly investigated, there is an increasing awareness about its ecological importance for its biodiversity, as fish nursery and for the recruitment of shallow water species. Along with coastal rocky cliffs, isolated coralligenous concretions emerging from muddy bottoms are typical structures of the Mediterranean Sea mesophotic zone. Coralligenous concretions at mesophotic depths in the South Tyrrhenian Sea were investigated to assess the role of these coralligenous oases in relation to the biodiversity of surrounding soft sediments. We show here that the complex structures of the coralligenous concretions at ca. 110 m depth influence the trophic conditions, the biodiversity and assemblage composition in the surrounding sediments even at considerable distances. Coral concretions not only represent deep oases of coral biodiversity but they also promote a higher biodiversity of the fauna inhabiting the surrounding soft sediments. Using the biodiversity of nematodes as a proxy of the total benthic biodiversity, a high turnover biodiversity within a 200 m distance from the coralligenous concretions was observed. Such turnover is even more evident when only rare taxa are considered and seems related to specific trophic conditions, which are influenced by the presence of the coralligenous structures. The presence of a high topographic complexity and the trophic enrichment make these habitats highly biodiverse, nowadays endangered by human activities (such as exploitation of commercial species such as *Corallium rubrum*, or trawling fisheries, which directly causes habitat destruction or indirectly causes modification in the sedimentation and re-suspension rates). We stress that the protection of the coralligenous sea concretions is a priority for future conservation policies at the scale of large marine ecosystems and that a complete census of these mesophotic oases of biodiversity should be a priority for future investigations in the Mediterranean Sea.

## Introduction

The Mediterranean Sea is a miniature ocean (0.82% of the global oceans' surface and 0.32% of global ocean volumes), which hosts approximately 17 000 species (ca 8% of the global marine biodiversity) and an extremely high percentage of endemic species [1]–[3]. While shallow water biodiversity and, more recently, deep-sea biodiversity have been intensively investigated [4], the mesophotic (twilight) zone remains among the less explored marine habitats of the entire Mediterranean Sea [5]–[7]. In oceanic regions at tropical latitudes, the twilight zone (characterized by the presence of the mesophotic reefs) is increasingly arousing attention for its peculiar environmental features and high habitat complexity, which support high levels of biodiversity [8].

The twilight zone of the Mediterranean Sea (typically 50–200/300 m water depth), shows the presence of typical coralligenous concretions. The coralligenous habitat is a hard substratum of biogenic origin, mainly produced by the accumulation of calcareous encrusting algae growing in dim light conditions [9], [10]. Due to their pre-eminent tridimensional architecture, the coralligenous ecosystems are characterized by the presence of a variety of microhabitats, they are inhabited by complex species assemblages and they host a rich associated fauna attracting numerous visiting species [10]. Among all the organisms inhabiting the coralligenous ecosystems, corals, gorgonians and sponges play an important ecological role in the pelagic–benthic transfer of energy and biomass and amplify the ecosystem's overall complexity [5], [11], [12]. In addition, these megafaunal taxa act as ecosystem engineers [13] and contribute to alter current flow velocity, stabilising soft substrata, increasing particle sedimentation and enhancing local accumulation of fine particles [14]. Due to their low growth rates and their limited capability to deal with large and/or abrupt environmental fluctuations, these organisms appear to be particularly vulnerable to natural [15] and human-driven anthropogenic impacts, including trawling and long-line fisheries, pollution and biological invasions [11], [12].

Along with coastal rocky cliffs, isolated coralligenous concretions emerging from muddy bottoms are typical structures of the Mediterranean Sea mesophotic zone [7]. The biodiversity of sessile faunal assemblages, inhabiting these coral oases, has been recently investigated [7], [11], however, the effects of these oases on surrounding soft sediments remains unveiled or unknown [5].

Metazoan meiofauna are considered a sensitive tool for investigating biodiversity patterns and structural and functional features of benthic marine ecosystems because of their ecological importance and the lack of larval dispersion [16]–[18]. Nematodes represent the numerically dominant taxon in marine sediments worldwide (typically up to more than 1 million individuals m^−2^) and they show high biodiversity (possibly they are hyper-diverse [19]); in addition their biodiversity displays patterns which reflect those of the total benthic biodiversity [4], [17].

Very little information is available on the role of habitat heterogeneity in modulating the biodiversity of soft-bottom sediments surrounding coral-associated habitats [20], [21]. For example, it has been shown that the meiofaunal abundance and biodiversity in proximity of forests of the gold coral *Savalia savaglia* are significantly higher than soft bottoms at the same depth but without corals. One of the possible explanations of this difference might be attributed to differences in the food availability [5].

In this study, we investigated the role of coral oases in influencing the biodiversity of the seafloor sediments surrounding and we also provide new suggestions for the protection of coralligenous concretions of the Gulf of Santa Eufemia, located in the Southern Tyrrhenian Sea (Western Mediterranean sea).

## Methods

### Ethics Statement

The ROV surveys and the sampling operations in the study area were conducted with regular permits (Aut. 79 ICRAM, R031250, Giu. 08– GE/DN7005; Aut. 96 ICRAM, 104 R090737, Lug. 08; MARISTAT 2008 P261430Z; Reggio Calabria Port Authority N°15 2008) released by the Italian Navy Hydrographic Institute and the Calabria Port Authorities.

### Study area and sampling

**Figure 1:**
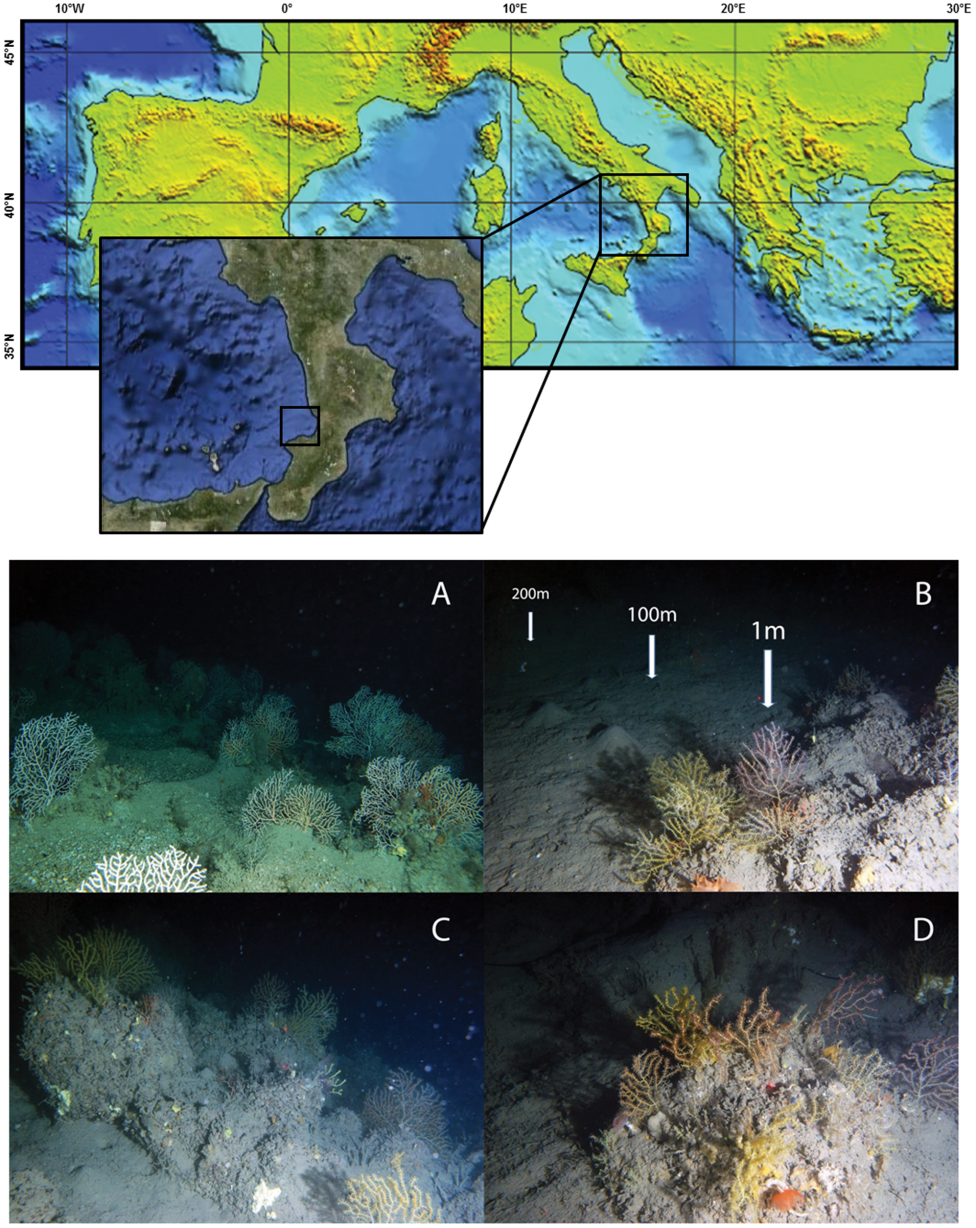
Location of the sampling area in the Santa Eufemia gulf, South Tyrrhenian sea (Western Mediterranean Sea). Coralligenous concretions in the Santa Eufemia gulf are also illustrated: A) concretions dominated by *Acanthogorgia* sp; B) schematic view of the sampling strategy (not in scale) in the soft bottoms surrounding the coralligenous concretions; C) and D) coralligenous concretions surrounded by soft bottom sediments.

This study was conducted in the Southern Tyrrhenian Sea (Western Mediterranean Sea), specifically in the Santa Eufemia Gulf (Fig. 1), at ca. 110 m depth. The area is characterized by the presence of numerous small rocky shoals distributed over gently sloping muddy grounds. Each shoal is composed of one or few major elevations (up to several m high) surrounded by mud and smaller, sparse rocks, partially covered by silt at the time of sampling [7]. These shoals host large concretions made by rich megabenthic communities dominated by coral species and many other taxa (sponges, cnidarians, molluscs and fishes) [7]. These isolated rocky concretions represent a good example of “roche du large” ecosystems [22] (literally, “offshore rocks”), which concentrate sessile macrofaunal biodiversity [7]. The shoal under scrutiny hosts the highest number of encrusting taxa in the study area [7].

Sediment sampling was conducted on 4–5th June 2008 on board of the R/V Astrea, by means of a box corer, after a ROV survey which allowed the localisation of the coralligenous concretions and the identification of the sessile megafauna inhabiting this habitat (Fig. 1) [7].

Samples were collected along three randomly chosen transects, each comprising three stations located at increasing distance from the coralligenous concretions (at 1, 100 and 200 m). The location, distance from the coralligenous concretions and water depth of each sampling station are reported in Table 1. At each station the box-corer was deployed three times. From each deployment the first centimetre of sediment was extracted using Plexiglas corers (internal diameter: 4.7 cm) and was immediately frozen at −20°C for further analyses of organic matter biochemical composition in the laboratory (within two weeks). From the same three deployments of the box-corer additional sediment subsamples were collected using Plexiglas corers (internal diameter: 3.6 cm). The top 1 cm of each sediment subsample was fixed with 4% buffered formalin and stained with Rose Bengal (0.5 gL^−1^) until meiofaunal analyses in the laboratory.

**Table 1:** Location, distance from the coralligenous concretions, water depth, concentration of biochemical compounds (total phytopigment, protein, carbohydrate, lipid and biopolymeric C) and nutritional quality (phytopigment and protein contribution to the bulk of biopolymeric C and the values of the protein to carbohydrate ratio) in the sediments at the investigated stations.

	Distance	Latitude (N)	Longitude (E)	Water depth	Total phytopigments		Proteins		Carbohydrates		Lipids		Biopolymeric C		Phytopigment: biopolymeric C ratio		Protein: biopolymeric C ratio		Protein: carbohydrate ratio	
	(m)			(m)	µg g^−1^	sd	mg g^−1^	sd	mg g^−1^	sd	mg g^−1^	sd	mgC g^−1^	sd	avg	sd	avg	sd	avg	sd
Transect 1	1	38° 44.41′	16° 05.49′	110	3.3	1.2	1.4	0.1	2.5	0.4	0.7	0.1	2.3	0.4	5.6	1.2	30.6	2.9	0.6	0.1
	100	38° 44.39′	16° 05.38′	113	6.5	2.5	3.8	0.3	2.1	0.4	0.7	0.1	3.3	0.4	7.7	2.2	56.3	1.7	1.8	0.2
	200	38° 44.37′	16° 05.32′	113	4.1	1.3	1.5	0.2	2.5	0.3	1.3	0.1	2.7	0.1	6.0	1.7	26.6	2.8	0.6	0.1
Transect 2	1	38° 44.41′	16° 05.46′	111	6.7	1.0	0.8	0.1	1.6	0.2	0.5	0.1	1.4	0.1	19.6	1.4	26.8	1.6	0.5	0.1
	100	38° 44.38′	16° 05.51′	115	3.4	0.6	1.1	0.1	1.7	0.3	0.4	0.0	1.4	0.2	9.5	0.6	38.2	2.3	0.7	0.1
	200	38° 44.35′	16° 05.57′	114	4.1	0.9	3.7	0.9	2.5	0.5	0.6	0.0	3.3	0.6	5.0	0.2	55.6	3.6	1.5	0.1
Transect 3	1	38° 44.42′	16° 05.45′	115	3.0	1.9	2.5	0.3	1.5	0.3	1.6	0.2	3.0	0.1	4.0	2.4	40.8	3.8	1.7	0.1
	100	38° 44.47′	16°05.45′	111	3.8	2.2	2.2	0.1	0.9	0.2	0.5	0.1	1.8	0.1	8.2	4.5	59.6	0.5	2.4	0.4
	200	38° 44.52′	16°05.45′	110	6.4	1.7	1.1	0.1	2.6	0.4	0.6	0.1	2.0	0.1	12.4	2.7	27.2	2.0	0.4	0.1

### Food availability and trophic conditions

Chloroplastic pigments (chlorophyll-a and phaeopigments) utilized as a proxy of the input of algal and microphytobenthic biomass were analyzed fluorometrically [23]. Total phytopigment concentrations were defined as the sum of chlorophyll-a and phaeopigment concentrations, and considered as an estimate of the organic material of algal origin, including the living (chlorophyll-a) and senescent/detrital (phaeopigment) fractions [24]. In the present study, we converted sediment phytopigment concentrations into carbon equivalents using a mean value of 40 µg C µg phytopigment^−1^ [24], [25].

The biochemical composition of sediment organic matter (protein, carbohydrate and lipid contents) was determined to assess the quantity and quality of the organic matter available to benthic consumers [24]. Protein, carbohydrate and lipid analyses were carried out according to Danovaro [23], and concentrations reported as albumin, glucose and tripalmitine equivalents, respectively. For the analysis of each biochemical class of organic compounds, blanks were made with the same sediment samples previously treated in a muffle furnace (450°C, 2 h). All biochemical analyses were carried out (n = 3) on the top 1 cm of the sediment. Protein, carbohydrate and lipid concentrations were converted into carbon equivalents using the conversion factors 0.49, 0.40 and 0.75 mg C mg^−1^, respectively and the sum of protein, carbohydrate and lipid carbon was referred as biopolymeric carbon (BPC) [24], [25].

For the purposes of the present study, we used the percentage contributions of phytopigment and protein to biopolymeric C concentrations and the values of the protein to carbohydrate ratio as descriptors of the aging and nutritional quality of sediment organic matter [24], [26].

### Meiofaunal assemblages

For meiofaunal extraction, sediment samples were sieved through a 1000-µm mesh, and a 20-µm mesh was used to retain the smallest organisms. The fraction remaining on the latter sieve was re-suspended and centrifuged three times with Ludox HS40 (diluted with water to a final density of 1.18 g cm^−3^) as reported in Danovaro [23]. All animals remaining in the surnatant were passed again through a 20 µm mesh net and, after staining with Rose Bengal, sorted under a stereomicroscope (x40 magnification) [23]. The rare taxa were defined as those taxa that represented each <1% of the total meiofaunal abundance in all investigated samples [27].

Meiofaunal biomass was assessed by bio-volumetric measurements for all specimens encountered. Nematode biomass was calculated from the biovolume, using the formula reported in [28] (V = L×W^2^×0.063×10^−5^, in which body length, L, and width, W, are expressed in mm). Body volumes of all other taxa were derived from measurements of body length (L, in mm) and width (W, in mm), using the formula V = L×W^2^×C, where C is the approximate conversion factor for each meiofaunal taxon [29]. Each body volume was multiplied by an average density (1.13 g cm^−3^) to obtain the biomass (mg DW:mg WW = 0.25; [30]) and the carbon content was considered to be 40% of the dry weight [29]. The biomass was expressed as µgC 10 cm^−2^.

### Nematode biodiversity

All nematodes from 0–1 cm layer of sediment from each independent replicate were mounted on slides, following the formalin-ethanol-glycerol technique to prevent dehydration [23], [31]. The nematodes were identified to species level according to the standard manuals [31]–[35] and the most recent literature dealing with new nematode genera and species. All of the unknown species were indicated as sp_1_, sp_2_, sp_3_,…sp_n_. The number of identified nematodes at each sampling station was reported in Table 2.

**Table 2:** Location, distance from the coralligenous concretions, water depth, meiofaunal communities abundance, biomass, number of identified nematodes and nematode diversity indexes (richness of meiofaunal higher taxa, nematode diversity indices: SR species richness; ES(100) expected species number for 100 individuals; H′^2^ Shannon's index; J′ species evenness; 1 – ITD index of trophic diversity; MI maturity index) in the sediments at the investigated stations.

	Distance	Latitude (N)	Longitude (E)	Water depth	Total meiofaunal abundance		Total meiofaunal biomass		Richness of taxa		N. identified nematodes	SR	ES100	H′^2^	J′	1-ITD	MI
	(m)			(m)	ind.10 cm^−2^	sd	µgC 10 cm^−2^	sd	avg	sd							
Transect 1	1	38° 44.41′	16° 05.49′	110	343.7	107.6	24.2	13.4	5.0	1.7	230	73	47.3	5.5	0.9	0.6	2.8
	100	38° 44.39′	16° 05.38′	113	590.0	277.2	33.7	22.9	7.0	2.0	211	73	50.5	5.7	0.9	0.7	2.8
	200	38° 44.37′	16° 05.32′	113	500.1	166.6	27.0	19.6	7.0	1.7	251	78	49.2	5.7	0.9	0.7	2.6
Transect 2	1	38° 44.41′	16° 05.46′	111	483.8	276.6	96.2	57.8	7.3	1.5	257	80	52.1	5.8	0.9	0.7	2.7
	100	38° 44.38′	16° 05.51′	115	132.6	70.2	13.2	22.6	3.0	1.7	75	37	37.0	4.9	0.9	0.6	2.7
	200	38° 44.35′	16° 05.57′	114	388.1	35.6	41.4	13.0	3.3	1.5	266	66	45.1	5.5	0.9	0.7	2.7
Transect 3	1	38° 44.42′	16° 05.45′	115	190.1	9.7	8.4	7.2	6.0	1.7	261	83	52.8	5.9	0.9	0.6	2.8
	100	38° 44.47′	16°05.45′	111	483.5	382.1	60.7	36.2	6.7	1.5	219	77	52.2	5.7	0.9	0.7	2.7
	200	38° 44.52′	16°05.45′	110	275.4	92.5	46.8	25.3	6.0	1.0	146	64	51.2	5.4	0.9	0.7	2.8

The nematode point α-diversity [36] was estimated using the species richness (SR), as the number of different species cumulatively retrieved from the three independent samplings at each sampling station. As the number of nematodes extracted differed among replicates and sampling stations, the expected number of species was also considered, which provides a standardisation of the values of the species richness according to the sample size. The expected number of species for a theoretical sample of 100 specimens, ES(100), was chosen to facilitate the comparisons among transects and distances.

The species diversity was also measured by the Shannon-Wiener index (H′, using log-base 2, expressed as H′^2^), and the evenness was measured by the Pielou's J index [37]. These indices were calculated from the sum of the individuals of the three replicates collected in each of the sampling stations, using the DIVERSE routine included in the PRIMER v6.0+ software [38].

We also measured the β-diversity, (i.e., turnover diversity) [36] between stations located at the different distances (i.e., 1, 100 and 200 m) as the percentage of the dissimilarity of nematode community species composition, calculated from resemblance matrices based on Bray-Curtis similarity (SIMPER, included in the PRIMER v6.0+ software).

The trophic composition of the nematode assemblages was defined according to Wieser [39]. Nematodes were divided into four groups, according to their buccal morphology: no buccal cavity or a fine tubular one (selective-bacterial feeders; 1A); large but unarmed buccal cavity (non-selective deposit feeders; 1B); buccal cavity with scraping tooth or teeth (epistrate or epigrowth-diatom feeders; 2A); and buccal cavity with large jaws (predators/omnivores; 2B). The Index of Trophic Diversity (ITD) was calculated as 1-ITD, where ITD  =  g_1_^2^+g_2_^2^+g_3_^2^…+g_n_^2^, g is the relative contribution of each trophic group to the total number of individuals, and n is the number of trophic groups [40]. For n = 4 (as in the present study), the 1-ITD ranges from 0.00 to 0.75.

The maturity index indicates the nematode assemblage maturity [41]. According to their life strategies, nematodes genera are classified as “colonizers” (r-strategists) or “persistent” (k-strategists), in a scale from 1 to 5 (c-p value, colonizer-persistent). To determine the colonisation strategies of the nematodes, the maturity index (MI) was calculated according to the weighted mean of the individual genus scores, as Σ ν (i) ƒ (i), where ν is the colonisers-persisters (c-p) value of the genus i [41], and ƒ (i) is the frequency of that genus.

### Statistical analyses

To assess differences between transects and distances, we applied either uni- or multivariate analyses of variance. All the statistical analyses were carried out using the same sampling design, considering two factors as main sources of variance: transect (T, random factor, 3 levels: 1, 2 and 3) and distance (D, fixed factor, 3 levels: 1, 100 and 200 m).

First, for meiofaunal abundance and biomass, richness of meiofaunal taxa and nematode diversity indexes (SR, ES100, H′^2^, J′, 1-ITD and MI), two-way univariate analyses of variance (ANOVA) were carried out. Prior to analyses, the normal distribution of the data was checked using the Shapiro-Wilk test, which is one of the most powerful tests of normality especially for small samples. Then, the homogeneity of variance was tested by means of the Cochrans' test and, when necessary, the data were appropriately transformed. For those data for which the transformation did not allow to obtain homogeneous variances, a more conservative level of significance was adopted [42]. When significant differences were observed between stations at increasing distance from the concretions, post-hoc comparison (Student-Newman-Kuels, SNK) tests were also applied. Since for the meiofaunal biomass the interaction Transect × Distance was significant, the SNK test was performed testing the factor “distance” separately for each transect. The Shapiro-Wilk test was carried out using the routine included in the XLSTAT software. ANOVA, Cochran's and SNK tests were carried out using the GMAV 5.0 software (University of Sidney).

A distance-based permutational multivariate analysis of variance (PERMANOVA) [43], [44] was also used to investigate variations in organic matter biochemical composition and nutritional quality. The analysis was based on Euclidean distances of previously normalized data, using 999 random permutations of the appropriate units [45], [46]. When significant differences were observed between stations at increasing distance from the concretions, pairwise comparisons were also performed. Prior to perform the PERMANOVA analysis, a PERMDISP test was also performed to exclude that pure spatial aggregation of the samples was causing the significant interaction. Bi-plots produced after a principal component analysis (PCA) were used to visualize differences among sampling stations in the sedimentary organic matter biochemical composition and nutritional quality.

Two-way SIMPER analyses were performed to assess the percentage dissimilarity in the meiofaunal and nematode community composition between transects and distances within the same transect, separately for i) the whole meiofaunal community, ii) the meiofaunal rare taxa community and iii) the nematode assemblages. The two-way SIMPER analyses were also performed to identify which among the investigated taxa/species was mostly responsible for the observed dissimilarities. A ranked matrix of Bray–Curtis similarities, constructed on previously square-root-transformed data, was used as input for this test. A two-way analysis of similarities (ANOSIM) was performed to test for the presence of statistical differences in meiofaunal taxonomic composition (both the whole and rare taxa communities) and nematode species composition between transects and distances within the same transect.

PERMANOVA, SIMPER, ANOSIM, PCA and PERMDISP analyses were performed using the routines included in the PRIMER 6+ software [38].

## Results

Data of phytopigment, protein, carbohydrate, lipid and biopolymeric C sedimentary contents, the values phytopigment and protein contribution to the bulk of biopolymeric C and the values of the protein to carbohydrate ratio, total meiofaunal abundance and biomass, richness of meiofaunal higher taxa, and nematode diversity indices are reported in Table 1 and Table 2.

All data dealing with meiofaunal taxa and nematode species identified in the present study are reported in Supporting information (Table S1 and Table S2, respectively).

### Food availability to benthic consumers

**Figure 2:**
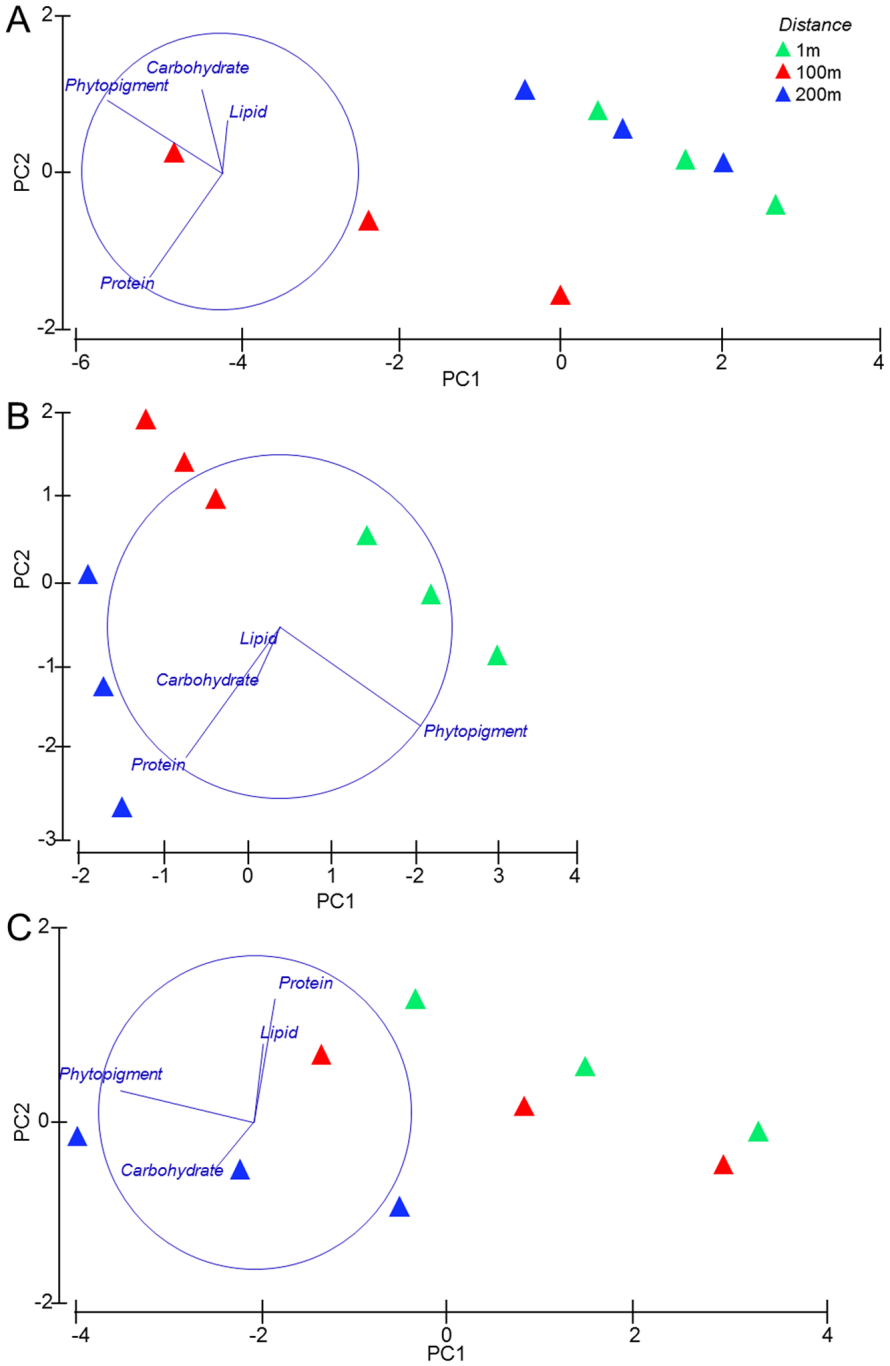
Output of the PCA analysis conducted on the sedimentary organic matter biochemical composition along the Transect 1 (A); Transect 2 (B) and Transect 3 (C).

**Figure 3:**
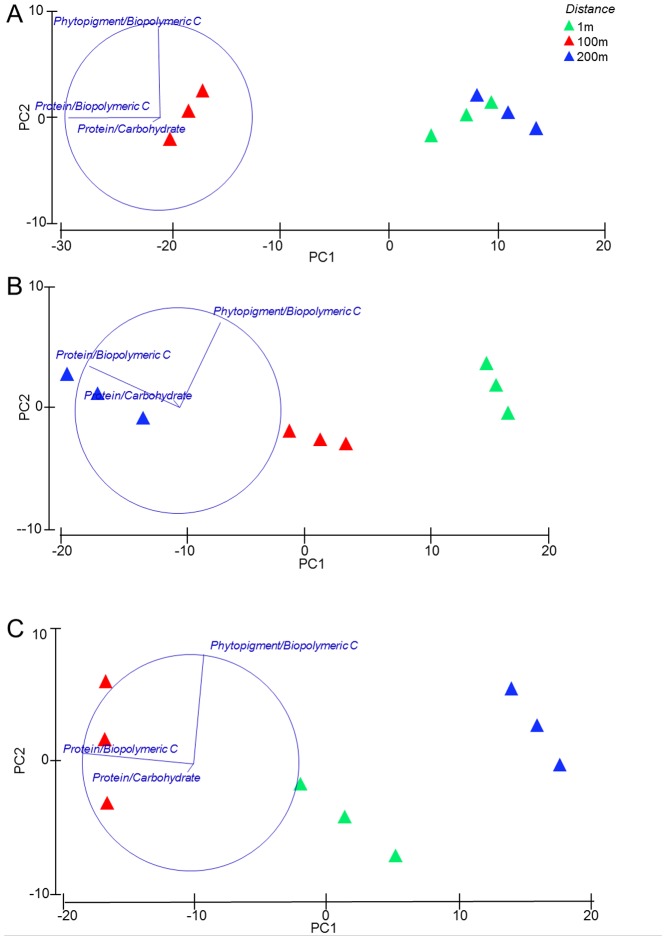
Output of the PCA analysis conducted on the sedimentary organic matter nutritional quality along the Transect 1 (A); Transect 2 (B) and Transect 3 (C).

The algal fraction of biopolymeric C ranged from 4 to 20% (Table 1). The protein fraction of biopolymeric C ranged from 27 to 60% (Table 1). The algal and protein fractions of biopolymeric C and the values of the protein to carbohydrate ratio varied significantly with increasing distance from the concretions (Table 1). The results of the PERMDISP test, conducted prior the PERMANOVA analysis, did not reveal differences in dispersion between the *a priori* groups (data not shown). The PERMANOVA tests showed that the interaction Transect × Distance had significant effects both on the biochemical composition and the nutritional quality of sedimentary organic matter, indicating that the biochemical composition of sedimentary organic matter varied with increasing distance from the concretions, but with different and variable patterns in each transect (Table 3). In particular, the pair wise tests indicated that the biochemical composition varied significantly among all distances along the Transect 2, and only between 1 and 100 m in the Transect 1 and between 1 and 200 m in the Transect 3 (Table 3; Fig. 2). The nutritional quality of the sedimentary organic matter varied significantly among all distances along all the investigated transects, except between 1 and 200 m in the Transect 1 (Table 3; Fig. 3).

**Table 3:** Output of the distance-based permutational multivariate analysis of variance (PERMANOVA) carried out on the biochemical composition and nutritional quality of sedimentary organic matter.

A)		Main test				
	Source	df	MS	Pseudo-F	P(MC)	ECV (%)
Biochemical composition	Transect	2	2.306	0.795	-	0.0
	Distance	2	4.792	0.227	-	0.0
	Transect × Distance	4	21.147	7.291	***	67.7
	Residual	18	2.9			32.3
	Total	26				
Nutritional quality	Transect	2	107.76	9.325	-	3.7
	Distance	2	882.2	1.138	-	4.1
	Transect × Distance	4	775.09	67.075	***	88.2
	Residual	18	11.556			4.0
	Total	26				

B)		Transect 1		Transect 2		Transect 3	
	Pairwise	t	P	t	P	t	P
Biochemical composition	1 vs 100	2.447	*	4.461	*	0.867	ns
	1 vs 200	0.979	ns	3.936	**	2.558	*
	100 vs 200	2.014	ns	3.113	*	1.996	ns
Nutritional quality	1 vs 100	10.611	**	8.436	**	5.207	**
	1 vs 200	1.53	ns	13.355	***	4.933	**
	100 vs 200	12.078	***	7.244	**	9.907	***

A) main test and B) pairwise comparisons. df  =  degree of freedom, MS  =  mean square, F  =  ANOVA F statistic, P  =  probability level. ***  =  P<0.001; **  =  P<0.01, *  =  P<0.01; ns  =  not significant. ECV  =  Estimated Components of Variance (%).

### Meiofaunal abundance and biomass

Total meiofaunal abundance and biomass in the sediments surrounding the coralligenous concretions are reported in Table 2. The two-way analyses of variance (ANOVA, Table S3) revealed only weak differences among the investigated transect and/or distances in the meiofaunal variables (ANOVA, ns; Table S3). Indeed, no significant differences were found in the total meiofaunal abundance among different transects and/or distances, while the total meiofaunal biomass displayed a significant effect of the Transect × Distance interaction (ANOVA, p<0.05). Pairwise comparisons analysis of the meiofaunal biomass in Transect 2, over the three investigated, displayed higher values at 1 m when compared to 100 m and 200 m only (ANOVA; p<0.05; Table S3).

### Biodiversity in sediments surrounding the coralligenous concretions

**Figure 4:**
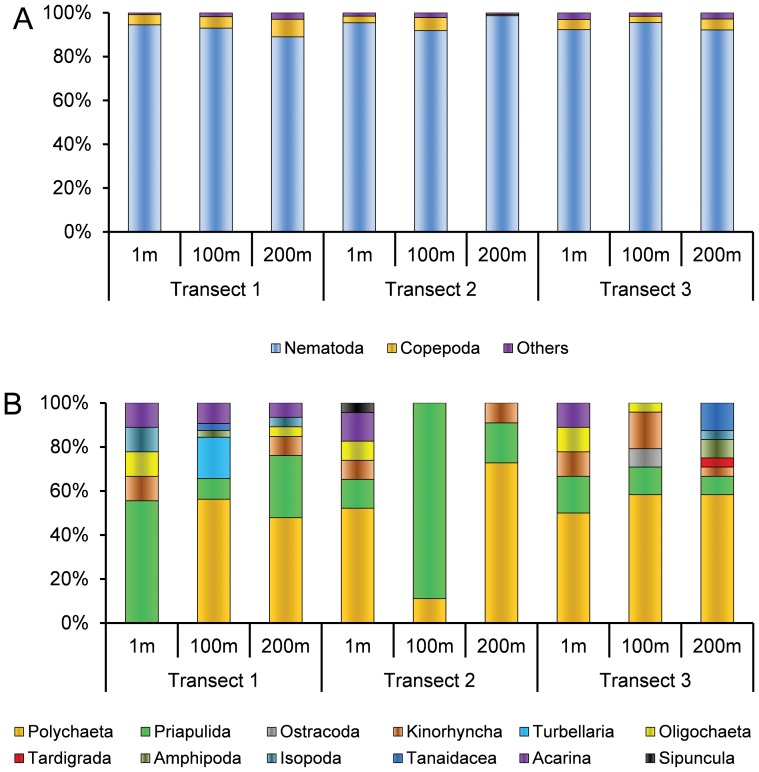
Community structure of the entire meiofaunal assemblage (A) and zoom on the rare taxa (B) the investigated stations.

The richness of meiofaunal higher taxa and nematode diversity indices (SR, ES(100), H′^2^, J′, 1-ITD and MI) are reported in Table 2. Overall, the meiofaunal communities were dominated by nematodes (89–99%) and copepods (0.4–8%) at all investigated stations (Fig. 4A), whereas the rare taxa communities (i.e., the community of taxa accounting each for <1% of the whole community) were characterized by different assemblages of taxa, depending on the investigated station (Fig. 4B).

**Figure 5:**
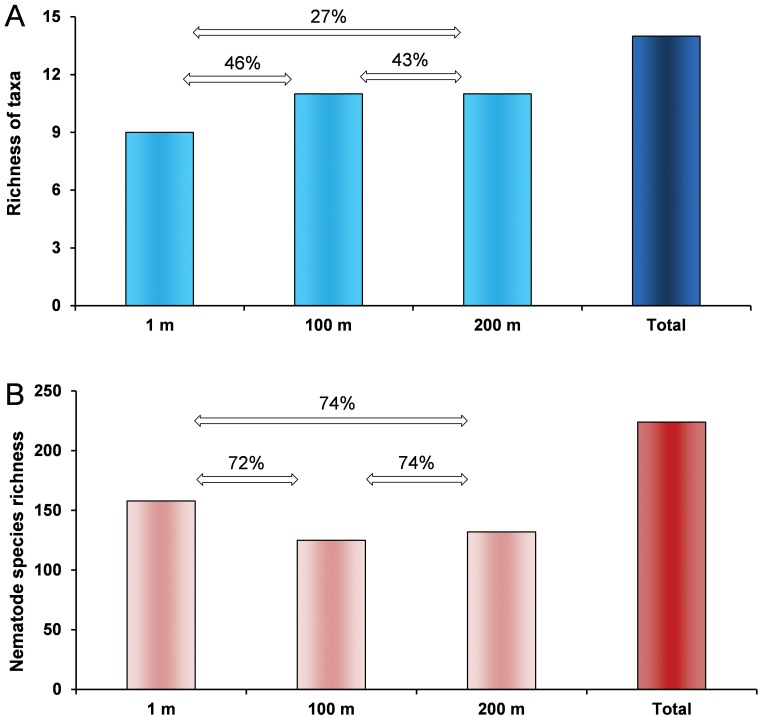
Richness of meiofaunal higher taxa (A) and nematode species richness (B) at each investigated distance from coralligenous concretions and in the overall investigated area. Dissimilarity (%) in the A) meiofaunal higher taxa and B) nematode species composition among different distances from the coralligenous concretions is also illustrated.

In Figure 5, the overall richness of meiofaunal taxa (Fig. 5A) and nematode species richness (Fig. 5B), found at each investigated distance, as well as the total richness of meiofaunal taxa and nematode species richness found in the study area, cumulatively from all the investigated stations, are illustrated. The overall % dissimilarity between different distances (e.g., cumulatively, between the stations at 1 m vs. the stations at 100 m) in the meiofaunal (Fig. 5A) and nematode (Fig. 5B) assemblages is also reported.

In particular, polychaetes, priapulids, kinorhynchs, oligochaetes, amphipods, isopods, tanaidaceans and acarina were found at all investigated distances from the coralligenous concretions, whereas sipuncula were exclusive at 1 m, ostracods and turbellarians at 100 m and tardigrada at 200 m.

We found 224 nematode species (over 1916 individuals examined), 25% of which were exclusive at the 1 m stations, 11% at the 100 m distance and 13% at the 200 m distance, whereas 51% of the species were in common among stations across the entire sampling transects.

The statistical analysis conducted on the richness of meiofaunal taxa and nematode diversity indexes (Table S4) revealed significant differences between the distances in the Transect 2 (ANOVA, p<0.05), with higher values at 1 and 200 m than 100 m (SNK, p<0.05), for almost all of the investigated indexes.

The ANOSIM analyses conducted separately for the: i) whole meiofaunal assemblage; ii) rare taxa and iii) nematode assemblages revealed significant differences between transects and distances (Table 4). In particular, significant differences were observed in the rare taxa taxonomic composition at Transect 1 (between stations at 1 and 100 m and between stations at 1 and 200 m; ANOSIM, p<0.05; Table 4) and in Transect 2 (between 1 and 100 m; ANOSIM, p<0.05; Table 4). The SIMPER analyses revealed that the dissimilarity between sampling distances ranged from 22 to 67% for the entire meiofaunal communities but it raised up to 84% for the rare taxa (Table 4). The SIMPER analyses revealed also that the taxa mostly responsible for the observed dissimilarities were nematodes, copepods and different assemblages of rare taxa (Table 4). It is worth noting that the dissimilarity of the meiofaunal assemblages composition between immediately adjacent sampling stations (i.e., 1 vs. 100 m and 100 vs. 200 m) was always high (Fig. 5). This is mostly due to the presence of different exclusive meiofaunal taxa at each investigated distance. Significant differences among transects and distances were also observed in the nematode assemblage composition (ANOSIM, p<0.05; Table 4). The SIMPER analyses revealed that the dissimilarity of the nematode species composition between distances ranged from 58 to 82% (Table 4).

**Table 4:** Output of the SIMPER and ANOSIM analyses carried out on meiofaunal community composition of the A) whole meiofaunal community and B) meiofaunal rare taxa community and C) nematode species assemblages.

			ANOSIM		SIMPER	
			R	P	% Dissimilarity	
A)	Transect 1 *vs* Transect 2		0.16	ns	37.99	Nematoda, Copepoda
	Transect 1 *vs* Transect 3		0.20	ns	32.17	Nematoda, Copepoda
	Transect 2 *vs* Transect 3		0.36	*	43.27	Nematoda
	Transect 1	1 m *vs* 100 m	0.04	ns	29.36	Nematoda, Copepoda
		1 m *vs* 200 m	0.04	ns	21.98	Nematoda, Copepoda
		100 m *vs* 200 m	0.19	ns	24.67	Nematoda, Copepoda
	Transect 2	1 m *vs* 100 m	0.19	ns	67.23	Nematoda
		1 m *vs* 200 m	0.11	ns	24.05	Nematoda
		100 m *vs* 200 m	0.48	ns	63.20	Nematoda
	Transect 3	1 m *vs* 100 m	0.33	ns	42.57	Nematoda
		1 m *vs* 200 m	0.33	ns	23.16	Nematoda, Polychaeta
		100 m *vs* 200 m	0.04	ns	38.60	Nematoda
B)	Transect 1 *vs* Transect 2		0.40	**	79.27	Polychaeta, Priapulida, Acarina, Kinorhyncha, Turbellaria
	Transect 1 *vs* Transect 3		0.03	ns	58.27	Polychaeta, Kinorhyncha, Priapulida, Acarina, Oligochaeta, Turbellaria, Tanaidacea
	Transect 2 *vs* Transect 3		0.04	ns	74.75	Polychaeta, Priapulida, Kinorhyncha, Tanaidacea, Oligochaeta, Acarina
	Transect 1	1 m *vs* 100 m	0.74	*	78.01	Polychaeta, Turbellaria, Acarina, Priapulida, Kinorhyncha, Oligochaeta
		1 m *vs* 200 m	0.89	*	83.66	Polychaeta, Priapulida, Kinorhyncha, Acarina
		100 m *vs* 200 m	0.07	ns	50.34	Polychaeta, Priapulida, Turbellaria, Kinorhyncha, Acarina, Oligochaeta
	Transect 2	1 m *vs* 100 m	1.00	*	76.89	Priapulida, Polychaeta, Acarina, Oligochaeta
		1 m *vs* 200 m	0.04	ns	66.11	Polychaeta, Acarina, Priapulida, Oligochaeta, Kinorhyncha
		100 m *vs* 200 m	0.56	ns	79.26	Priapulida, Polychaeta
	Transect 3	1 m *vs* 100 m	0.037	ns	55.08	Polychaeta, Kinorhyncha, Ostracoda, Oligochaeta
		1 m *vs* 200 m	0.093	ns	70.72	Polychaeta, Tanaidacea, Priapulida, Kinorhyncha, Tardigrada, Amphipoda, Oligochaeta
		100 m *vs* 200 m	0.019	ns	62.23	Polychaeta, Kinorhyncha, Tanaidacea, Priapulida, Ostracoda, Amphipoda, Tardigrada
C)	Transect 1 *vs* Transect 2		0.51	*	72.48	*Richtersia sp1, Setosabatieria sp5, Richtersia sp8*
	Transect 1 *vs* Transect 3		0.80	**	81.36	*Richtersia sp1, Halalaimus filicaudatus, Leptolaimus sp1*
	Transect 2 *vs* Transect 3		0.49	*	69.79	*Elzalia sp1, Richtersia sp8, Graphonema sp1*
	Transect 1	1 m *vs* 100 m	0.04	ns	69.41	*Richtersia sp1, Sabatieria sp1, Leptolaimus sp1*
		1 m *vs* 200 m	1.00	**	81.85	*Leptolaimus sp1, Richtersia sp1, Microlaimus sp1*
		100 m *vs* 200 m	0.56	*	76.21	*Microlaimus sp1, Setosabatieria sp5, Aegialoalaimus sp1*
	Transect 2	1 m *vs* 100 m	0.11	*	58.83	*Terschellingia sp4, Pierrickia sp1, H. filicaudatus*
		1 m *vs* 200 m	0.41	*	58.23	*Aegialoalaimus sp4, Sabatieria sp1, Richtersia sp5*
		100 m *vs* 200 m	0.56	**	59.30	*Pierrickia sp1, Aegialoalaimus sp4, Sabatieria sp1*
	Transect 3	1 m *vs* 100 m	0.41	ns	64.46	*Graphonema sp1, Richtersia sp8, Pierrickia sp1*
		1 m *vs* 200 m	0.33	*	75.99	*Desmodora sp9, Richtersia sp8, Hopperia sp5*
		100 m *vs* 200 m	0.22	*	73.06	*Graphonema sp1, Desmodora sp9, Linhystera sp1*

## Discussion

The mesophotic habitats (also called “twilight” zone) are among the least-investigated marine ecosystems. Nonetheless, the few available results suggest that mesophotic habitats could have a major relevance on the functioning of both shallow and deep-sea environments [5], [47]. So far, investigations conducted in the mesophotic zone of the Mediterranean Sea have focused on the megafauna colonizing the coralligenous concretions [6], [7], [11]. These studies have proven that bio-constructor organisms (as gold corals or deep-water corals), thanks to the complex structure created by their large and hard skeletons, promote benthic biodiversity of hard bottoms by increasing the spatial heterogeneity of the substrate [5], [8], [48], [49]. Recent studies also demonstrated that these corals are able to promote biodiversity even when dead (e.g., coral rubbles), by creating novel substrates [20], [50]. These complex ecosystems are severely threatened by a variety of anthropogenic (e.g., trawling fisheries) and natural disturbances (e.g., climate-driven episodic events, such as dense shelf water cascading and temperature anomalies [15], [51].

Recent studies conducted in the twilight zone of the Mediterranean Sea, revealed that sediments surrounding mesophotic coral forests, when compared to the surrounding bare sediments, are characterised by significantly higher meiofaunal abundance and diversity [5]. In addition, cold water coral concretions and coral rubble mats are hot spots of biodiversity of metazoans and prokaryotic life [50], [52]. In contrast with those studies, we show here that the presence of coralligenous concretions can have a certain effect on the meiofaunal communities inhabiting the surrounding coral-free sediments, but also that the observed effects are more evident on meiofaunal diversity and community composition rather than on their abundance or biomass. Overall, only in one of the three investigated transects, total meiofaunal biomass and richness of taxa decreased progressively with increasing distance from the concretions, indicating that sources of variability other than the mere presence of coralligenous concretions were responsible for the distribution of meiofauna around the concretions themselves.

Most often, studies dealing with patterns of meiofaunal biodiversity have been focused on nematodes, copepods and polychaetes, due to the fact that such taxa are the most ubiquitous and resistant to different environmental disturbances [17]. Nevertheless, very recent investigations have demonstrated that the analysis of rare meiofaunal taxa (i.e. those taxa representing each <1% of total meiofaunal abundance) can provide additional insights to discriminate the effects generated by different sources of variability including spatial heterogeneity and organic pollution on meiofaunal community structure [18], [27]. Indeed, even though meiofaunal rare taxa seemed to be affected by very limited sample sizes, limited spatial coverage of sampling and patchy distributions of the meiofaunal assemblages, they provide useful information on the differences between meiofaunal assemblages in different environmental conditions [18], [27].

Here, we show that the effects of the presence of coralligenous concretions are more clearly detected if the analysis is carried out focusing on those meiofaunal taxa each representing <1% of the total meiofaunal abundance (i.e., rare taxa) [18], [53]. Indeed, when we consider the rare meiofaunal taxa only (i.e., excluding nematodes and copepods), the dissimilarities between stations at increasing distance from the coral concretion are enhanced (from 67 to 84%).

The presence of rare taxa (mostly juveniles of macro-megafaunal species) is important to assess the success of recruitment and the rare taxa are also indicators of the suitability of the substrate as nursery for several bentho-nekton species. In addition, nematodes and copepods, representing cumulatively the highest fraction of the meiofaunal assemblages, were responsible for consistently and substantially higher dissimilarity between stations when considering the entire meiofaunal assemblages. However, when we excluded from the analysis these taxa, which could hide differences between assemblages due to their dominance, we found out that different pools of rare taxa were responsible for the observed dissimilarity among stations (Table 4). This result confirms our hypothesis that the presence of the coralligenous concretions under scrutiny was not the unique source of variability in the composition of meiofaunal assemblages of coral-free soft sediments surrounding the coralligenous concretions. However, the high level of dissimilarity between adjacent distances found in the meiofaunal assemblages composition, due to the presence of exclusive meiofaunal taxa at each investigated distance, indicates that the presence of coralligenous concretions can anyway contribute to enhance the overall biodiversity of the surrounding soft bottoms seascape (up to 12 taxa found in this study, Fig. 5). They apparently attract some exclusive taxa (e.g., sipuncula) not encountered in the surrounding soft sediments at 100 or 200 m distance from the concretions. These results let us hypothesizing that the presence of coralligenous concretions may enhance the habitat heterogeneity in its close proximity, enhancing the overall biodiversity of the surrounding soft sediment seascape area, as previously described for areas characterized by other coralligenous concretions [49]. Previous studies reported that the variability in the biodiversity associated to coralligenous assemblages could be very high also at the very small spatial scale (i.e. within few meters) [54].

Also studies conducted in the deep sea reported that agglutinating protozoans appear to be a significant source of enhanced trophic resources and heterogeneity on the deep-sea floor, thus structuring metazoan (meio- and macrofauna) assemblages [55]. Also, small deep-sea sponges have been documented to be biogenic structures, enhancing the substrate heterogeneity and thus influencing the nematode biodiversity in surrounding sediments [21].

Recent studies conducted in the Mediterranean Sea twilight zone have highlighted the presence of black corals and gorgonians forming important complex habitats hosting a rich associated fauna and attracting numerous species of commercial interest [11]. However, previous studies related to the associated fauna were typically focused on macro- or megafauna [6], [7], [10], whereas information dealing with the biodiversity of smaller metazoans inhabiting the surrounding sediments are very scant. Here, by comparing nematode assemblages at different distances from the coralligenous concretions, we report the presence of high values of beta-diversity (i.e. turnover diversity) within a few hundreds of meters. These values indicate that different sampling sites are, to a large extent, colonized by different species assemblages. Within the very small investigated area (i.e., within a radius of 200 meters from the coralligenous concretions), only 51% of the nematode species were in common to all investigated distances, whereas at each site 11–25% of the species encountered were exclusive of the specific distance from the concretion (1, 100 or 200 m). These findings explain the huge biodiversity associated to this mesophotic habitat (224 species only for nematodes).

The influence of mesophotic coralligenous concretions on the twilight zone ecosystem processes as well as their biodiversity are still not fully understood [56]. Altogether, the results of this study point out that in the mesophotic zone the presence of engineering species and the complex structure created by the coralligenous concretions can enhance the overall biodiversity also in surrounding soft sediments, by promoting β-diversity (more than α-diversity). We also suggest that these results are driven by the important colonization by large ecosystem engineers of the hard substrates and by the effects of these complex structures on local hydrodynamism, which result in a locally enhanced and aggregated distribution of the food resources. These factors coupled with the high quality and diversified origin of the biopolymeric pools has an important effect on the turnover biodiversity and presence of exclusive benthic species.

As described in previous studies the quantity and composition of material settling down to the mesophotic zone has primarily terrestrial origin [5], [57], [58]. The concentrations of biopolymeric C in soft sediments in the twilight zone without coralligenous concretions are typically lower than those in close proximity to coral forests [5]. Here we show that the effect of coralligenous concretions is much wider than previously hypothesized. The analysis of mesophotic habitats of the Gulf of S. Eufemia showed that food quantity and quality of sedimentary organic matter were significantly influenced by the presence of coralligenous concretions. The effects were not consistent all around the concretions, as their complex structure had most likely an effect on the bottom currents and turbulent dynamics, which, in turn, influence the deposition of organic particles on the surrounding sediments. The effect of coral forests on the bottom current and small scale hydrodynamic and sedimentological processes has been already documented [14], and this partially explains why the amount and quality of the food available to benthic consumers matter displayed peaks either in close proximity of the coralligenous concretions, either at intermediate distance or at 200 m distance from the structure, depending on the transect considered. These results suggest that the effects observed at very small spatial scales (cm-m) related to isolated coral colonies in the mesophotic zone of the Ligurian Sea [5] are extended over a significantly larger spatial scale in the case of the coralligenous concretions of the S. Eufemia Gulf. We could infer that the effects of mesophotic coral and coralligenous concretions on the trophic status of surrounding soft sediments is dependent upon the size of the concretions, and, that the extent and direction of the observed trophic effects decreases with increasing size of the concretions. Although this hypothesis deserves further investigations, these findings suggest that coralligenous concretions could have important implications for the functioning of the entire twilight zone, by influencing the amount and distribution of the food resources available in the sediments.

Our results highlight that coralligenous concretions are of primary importance for their role in the functioning and in the maintenance of high biodiversity levels in the mesophotic ecosystems and thus deserve appropriate conservation strategies, which at present are not envisaged by policy makers. Given the poor knowledge on the distribution and extension of these habitats, we stress the need of investing in the production of detailed habitat mapping of the coralligenous concretions interspersed in soft bottom seascapes throughout the twilight zone of the Mediterranean Sea.

## Supporting Information

Table S1 **Meiofaunal taxa identified in the present study.**
(DOCX)

Table S2 **Nematode species identified in the present study.**
(DOCX)

Table S3 **Output of two-way ANOVAs carried out on meiofaunal abundance and biomass.** df  =  degree of freedom, MS  =  mean square, F  =  ANOVA F statistic, P  =  probability level. *  =  P<0.05, ns  =  not significant.
(DOCX)

Table S4 **Output of two-way ANOVAs carried out on richness of meiofaunal taxa and nematode diversity indexes.** df  =  degree of freedom, MS  =  mean square, F  =  ANOVA F statistic, P  =  probability level. *  =  P<0.05, ns  =  not significant. SR  =  species richness, H  =  Shannon-Wiener index, J  =  Pielou's index, ITD  =  index of trophic diversity, MI  =  maturity index.
(DOCX)
