# Supplementary material for: High Meiofaunal and Nematodes Diversity around Mesophotic Coral Oases in the Mediterranean Sea
Source: PLoS One. 2013 Jun 18;8(6):e66553. doi: 10.1371/journal.pone.0066553 (PMC3688901; doi:10.1371/journal.pone.0066553)
Supplement: Table S1 — Meiofaunal taxa identified in the present study. (DOCX) [file pone.0066553.s001.docx]

**Appendix S1**

**Table S1** Meiofaunal taxa identified in the present study.

|  | **Distance** | **Nematoda** | **Copepoda** | **Polychaeta** | **Priapulida** | **Ostracoda** | **Kinorhyncha** | **Turbellaria** | **Oligochaeta** | **Tardigrada** | **Amphipoda** | **Isopoda** | **Tanaidacea** | **Acarina** | **Sipuncula** |
| --- | --- | --- | --- | --- | --- | --- | --- | --- | --- | --- | --- | --- | --- | --- | --- |
|  | **(m)** | **%** | **%** | **%** | **%** | **%** | **%** | **%** | **%** | **%** | **%** | **%** | **%** | **%** | **%** |
| **Transect 1** | **1** | 94.49 | 4.66 | 0.00 | 0.48 | 0.00 | 0.10 | 0.00 | 0.10 | 0.00 | 0.00 | 0.10 | 0.00 | 0.10 | 0.00 |
|  | **100** | 92.99 | 5.23 | 1.00 | 0.17 | 0.00 | 0.00 | 0.33 | 0.00 | 0.00 | 0.06 | 0.00 | 0.06 | 0.17 | 0.00 |
|  | **200** | 89.03 | 7.97 | 1.44 | 0.85 | 0.00 | 0.26 | 0.00 | 0.13 | 0.00 | 0.00 | 0.13 | 0.00 | 0.20 | 0.00 |
| **Transect 2** | **1** | 95.41 | 3.04 | 0.81 | 0.20 | 0.00 | 0.14 | 0.00 | 0.14 | 0.00 | 0.00 | 0.00 | 0.00 | 0.20 | 0.07 |
|  | **100** | 91.87 | 5.91 | 0.25 | 1.97 | 0.00 | 0.00 | 0.00 | 0.00 | 0.00 | 0.00 | 0.00 | 0.00 | 0.00 | 0.00 |
|  | **200** | 98.65 | 0.42 | 0.67 | 0.17 | 0.00 | 0.08 | 0.00 | 0.00 | 0.00 | 0.00 | 0.00 | 0.00 | 0.00 | 0.00 |
| **Transect 3** | **1** | 92.27 | 4.64 | 1.55 | 0.52 | 0.00 | 0.34 | 0.00 | 0.34 | 0.00 | 0.00 | 0.00 | 0.00 | 0.34 | 0.00 |
|  | **100** | 95.54 | 2.84 | 0.95 | 0.20 | 0.14 | 0.27 | 0.00 | 0.07 | 0.00 | 0.00 | 0.00 | 0.00 | 0.00 | 0.00 |
|  | **200** | 92.17 | 4.98 | 1.66 | 0.24 | 0.00 | 0.12 | 0.00 | 0.00 | 0.12 | 0.24 | 0.12 | 0.36 | 0.00 | 0.00 |
