# Supplementary material for: High Meiofaunal and Nematodes Diversity around Mesophotic Coral Oases in the Mediterranean Sea
Source: PLoS One. 2013 Jun 18;8(6):e66553. doi: 10.1371/journal.pone.0066553 (PMC3688901; doi:10.1371/journal.pone.0066553)
Supplement: Table S2 — Nematode species identified in the present study. (DOCX) [file pone.0066553.s002.docx]

**Appendix S2**

**Table S2** Nematode species identified in the present study.

| **B)** | **Transect 1** | | |  | **Transect 2** | | |  | **Transect 3** | | |
| --- | --- | --- | --- | --- | --- | --- | --- | --- | --- | --- | --- |
|  | **1 m** | **100 m** | **200 m** |  | **1 m** | **100 m** | **200 m** |  | **1 m** | **100 m** | **200 m** |
|  | **%** | **%** | **%** |  | **%** | **%** | **%** |  | **%** | **%** | **%** |
| *Acantholaimus filicaudatus* | 0.0 | 0.5 | 0.0 |  | 0.0 | 0.0 | 0.0 |  | 0.0 | 0.0 | 0.0 |
| *Acantholaimus macramphis* | 0.0 | 0.5 | 1.6 |  | 0.0 | 0.0 | 0.0 |  | 0.0 | 0.0 | 0.0 |
| *Acantholaimus microdontus* | 0.4 | 0.0 | 0.0 |  | 0.0 | 0.0 | 0.0 |  | 0.0 | 0.0 | 0.0 |
| *Acantholaimus sp1* | 0.0 | 0.9 | 2.4 |  | 0.8 | 0.0 | 0.0 |  | 0.0 | 0.9 | 6.8 |
| *Acantholaimus sp2* | 0.0 | 0.0 | 0.0 |  | 0.0 | 0.0 | 0.0 |  | 0.4 | 0.0 | 0.0 |
| *Acantholaimus sp3* | 0.0 | 0.0 | 0.0 |  | 0.4 | 0.0 | 0.0 |  | 0.0 | 0.0 | 0.0 |
| *Acantholaimus sp9* | 0.0 | 0.0 | 0.0 |  | 0.0 | 0.0 | 0.0 |  | 0.0 | 0.5 | 0.0 |
| *Acantholaimus sp14* | 0.0 | 0.0 | 0.0 |  | 0.0 | 0.0 | 0.0 |  | 0.0 | 0.0 | 0.7 |
| *Acantholaimus sp17* | 0.0 | 0.0 | 0.4 |  | 0.0 | 0.0 | 0.0 |  | 0.0 | 0.0 | 0.0 |
| *Acantholaimus sp19* | 0.0 | 0.9 | 0.4 |  | 0.0 | 0.0 | 0.4 |  | 0.0 | 0.0 | 0.7 |
| *Adoncholaimus sp1* | 0.0 | 0.0 | 0.8 |  | 1.2 | 0.0 | 0.4 |  | 0.0 | 0.9 | 0.7 |
| *Adoncholaimus sp2* | 0.4 | 0.0 | 0.0 |  | 0.0 | 0.0 | 0.0 |  | 0.0 | 0.0 | 0.0 |
| *Aegialoalaimus sp1* | 0.0 | 0.5 | 5.6 |  | 2.3 | 0.0 | 1.9 |  | 1.9 | 3.7 | 0.7 |
| *Aegialoalaimus sp2* | 0.0 | 0.0 | 0.0 |  | 0.4 | 0.0 | 0.0 |  | 0.8 | 0.0 | 0.0 |
| *Aegialoalaimus sp3* | 0.0 | 0.0 | 0.0 |  | 0.4 | 0.0 | 0.0 |  | 0.0 | 0.0 | 0.0 |
| *Aegialoalaimus sp4* | 0.0 | 0.0 | 0.0 |  | 2.3 | 0.0 | 4.9 |  | 0.8 | 0.5 | 0.7 |
| *Amphimonhystera sp1* | 0.4 | 0.0 | 0.0 |  | 0.0 | 0.0 | 0.0 |  | 0.0 | 0.5 | 0.0 |
| *Amphimonhystera sp2* | 0.0 | 0.0 | 0.8 |  | 0.0 | 0.0 | 0.0 |  | 0.4 | 0.5 | 0.0 |
| *Amphimonhystera sp3* | 0.0 | 0.0 | 0.0 |  | 0.0 | 0.0 | 0.0 |  | 0.0 | 0.9 | 0.0 |
| *Amphimonhystrella sp1* | 2.6 | 0.9 | 0.4 |  | 0.8 | 0.0 | 0.0 |  | 0.8 | 0.0 | 0.0 |
| *Amphimonhystrella sp2* | 0.0 | 0.0 | 0.0 |  | 0.0 | 4.0 | 0.4 |  | 0.0 | 0.0 | 0.0 |
| *Amphimonhystrella sp3* | 0.0 | 0.5 | 0.0 |  | 0.0 | 0.0 | 0.0 |  | 0.0 | 0.0 | 0.0 |
| *Amphimonhystrella sp7* | 0.0 | 0.0 | 0.4 |  | 0.0 | 0.0 | 0.0 |  | 0.0 | 0.0 | 0.0 |
| *Amphimonhystrella sp8* | 0.0 | 0.0 | 0.0 |  | 0.0 | 1.3 | 0.0 |  | 0.0 | 0.0 | 0.0 |
| *Anoplostoma sp1* | 1.3 | 0.0 | 1.6 |  | 0.8 | 1.3 | 0.0 |  | 0.0 | 0.0 | 0.0 |
| *Antomicron sp1* | 0.4 | 0.0 | 0.0 |  | 0.0 | 0.0 | 0.0 |  | 0.0 | 0.0 | 0.0 |
| *Antomicron sp3* | 0.9 | 0.0 | 0.0 |  | 0.0 | 0.0 | 0.0 |  | 0.0 | 0.0 | 0.0 |
| *Bathyeurystomina sp1* | 0.0 | 0.0 | 0.0 |  | 0.0 | 0.0 | 0.0 |  | 1.1 | 0.0 | 0.0 |
| *Calligyrus sp1* | 0.0 | 1.4 | 0.4 |  | 0.0 | 1.3 | 0.0 |  | 0.0 | 0.0 | 0.0 |
| *Calyptronema sp1* | 0.9 | 0.5 | 0.0 |  | 0.0 | 0.0 | 0.0 |  | 0.0 | 0.0 | 0.0 |
| *Campylaimus sp1* | 2.2 | 2.4 | 2.0 |  | 2.7 | 2.7 | 3.4 |  | 0.4 | 2.3 | 0.0 |
| *Campylaimus sp3* | 0.0 | 0.0 | 0.0 |  | 0.4 | 0.0 | 0.0 |  | 0.0 | 0.0 | 0.0 |
| *Cephalanticoma sp1* | 0.0 | 0.0 | 0.0 |  | 0.0 | 2.7 | 1.5 |  | 0.4 | 0.0 | 0.7 |
| *Cephalanticoma sp2* | 0.0 | 0.0 | 0.0 |  | 1.9 | 0.0 | 0.0 |  | 0.4 | 0.0 | 0.0 |
| *Cervonema sp1* | 0.4 | 0.0 | 0.0 |  | 0.0 | 0.0 | 0.0 |  | 0.0 | 0.0 | 0.0 |
| *Cervonema sp2* | 0.0 | 0.0 | 1.2 |  | 3.9 | 2.7 | 2.3 |  | 1.1 | 0.9 | 1.4 |
| *Chaetonema sp1* | 0.0 | 0.0 | 0.4 |  | 0.0 | 0.0 | 0.0 |  | 0.0 | 0.0 | 0.0 |
| *Chromadorella sp1* | 0.0 | 0.5 | 0.0 |  | 0.0 | 0.0 | 0.0 |  | 0.0 | 0.0 | 0.0 |
| *Chromadorella sp2* | 0.0 | 0.0 | 1.2 |  | 0.0 | 0.0 | 0.0 |  | 0.0 | 0.0 | 0.0 |
| *Chromadorita sp1* | 0.0 | 0.0 | 0.0 |  | 0.4 | 0.0 | 0.0 |  | 0.0 | 0.0 | 0.0 |
| *Cobbia sp1* | 0.0 | 0.0 | 0.4 |  | 0.0 | 0.0 | 0.0 |  | 0.0 | 0.0 | 0.0 |
| *Comesoma sp1* | 0.0 | 0.5 | 0.0 |  | 0.0 | 0.0 | 0.0 |  | 0.0 | 0.0 | 0.0 |
| *Crenopharynx sp1* | 0.0 | 1.4 | 0.8 |  | 0.4 | 0.0 | 1.1 |  | 0.0 | 0.5 | 2.1 |
| *Crenopharynx sp2* | 0.0 | 0.0 | 0.0 |  | 0.0 | 0.0 | 0.0 |  | 0.0 | 0.5 | 0.0 |
| *Crenopharynx sp3* | 0.9 | 0.0 | 0.0 |  | 0.4 | 0.0 | 0.0 |  | 1.1 | 0.0 | 0.0 |
| *Cyartonema sp1* | 0.0 | 0.0 | 0.0 |  | 0.0 | 0.0 | 0.0 |  | 0.4 | 0.0 | 0.0 |
| *Cyatholaimus sp1* | 0.4 | 0.0 | 0.0 |  | 0.0 | 0.0 | 0.8 |  | 0.0 | 0.0 | 0.0 |
| *Cyatholaimus sp2* | 1.3 | 0.0 | 0.0 |  | 0.0 | 0.0 | 0.0 |  | 0.0 | 0.0 | 0.0 |
| *Desmodora sp1* | 1.3 | 0.5 | 0.0 |  | 0.0 | 0.0 | 0.8 |  | 0.0 | 0.9 | 1.4 |
| *Desmodora sp4* | 0.0 | 0.0 | 0.0 |  | 0.4 | 0.0 | 0.0 |  | 0.0 | 0.0 | 0.0 |
| *Desmodora sp5* | 0.0 | 0.0 | 0.0 |  | 0.8 | 0.0 | 0.0 |  | 1.9 | 0.0 | 0.7 |
| *Desmodora sp7* | 0.0 | 0.0 | 0.8 |  | 0.8 | 0.0 | 1.9 |  | 0.8 | 0.9 | 0.7 |
| *Desmodora sp8* | 0.0 | 0.0 | 0.0 |  | 0.4 | 0.0 | 0.4 |  | 0.8 | 0.5 | 2.1 |
| *Desmodora sp9* | 0.0 | 0.0 | 0.0 |  | 0.0 | 0.0 | 0.0 |  | 0.0 | 0.0 | 10.3 |
| *Desmodora sp10* | 0.0 | 0.0 | 0.0 |  | 0.4 | 0.0 | 0.0 |  | 0.0 | 0.0 | 0.0 |
| *Desmodorella sp1* | 0.0 | 0.0 | 0.0 |  | 0.0 | 0.0 | 0.0 |  | 0.4 | 0.0 | 0.0 |
| *Desmogerlachia sp1* | 0.0 | 0.0 | 0.0 |  | 0.0 | 0.0 | 0.0 |  | 0.0 | 0.9 | 0.7 |
| *Desmolorenzenia sp1* | 0.0 | 0.5 | 0.4 |  | 1.2 | 0.0 | 1.1 |  | 0.0 | 0.5 | 0.7 |
| *Desmolorenzenia sp5* | 0.0 | 0.0 | 0.0 |  | 0.0 | 1.3 | 0.0 |  | 0.0 | 0.0 | 0.0 |
| *Desmoscolex sp1* | 4.3 | 5.2 | 1.6 |  | 1.9 | 5.3 | 1.9 |  | 3.8 | 3.2 | 4.1 |
| *Desmoscolex sp2* | 3.0 | 0.5 | 0.0 |  | 1.9 | 1.3 | 1.1 |  | 0.4 | 0.0 | 0.0 |
| *Desmoscolex sp3* | 0.0 | 0.0 | 0.0 |  | 0.0 | 0.0 | 0.4 |  | 0.0 | 0.0 | 0.0 |
| *Desmoscolex sp4* | 0.0 | 0.5 | 1.2 |  | 0.8 | 0.0 | 0.0 |  | 0.4 | 0.5 | 0.0 |
| *Desmoscolex sp9* | 0.0 | 0.0 | 0.0 |  | 0.0 | 1.3 | 1.5 |  | 3.1 | 0.9 | 0.7 |
| *Desmoscolex sp10* | 0.0 | 0.0 | 0.0 |  | 0.4 | 0.0 | 0.0 |  | 0.4 | 0.0 | 0.0 |
| *Dichromadora sp1* | 0.9 | 1.4 | 2.8 |  | 2.3 | 0.0 | 0.0 |  | 0.4 | 0.0 | 0.0 |
| *Dichromadora sp3* | 0.0 | 0.5 | 0.0 |  | 0.0 | 0.0 | 0.0 |  | 0.0 | 0.0 | 0.0 |
| *Diplolaimella sp1* | 0.0 | 0.0 | 0.4 |  | 0.0 | 0.0 | 0.0 |  | 0.0 | 0.0 | 0.0 |
| *Diplopeltis sp7* | 0.0 | 0.0 | 0.4 |  | 0.0 | 0.0 | 0.0 |  | 0.0 | 0.0 | 0.0 |
| *Diplopeltoides sp1* | 0.9 | 0.9 | 1.2 |  | 1.2 | 1.3 | 0.0 |  | 0.0 | 0.0 | 0.0 |
| *Diplopeltoides sp2* | 0.0 | 0.9 | 0.4 |  | 0.0 | 0.0 | 0.0 |  | 0.0 | 0.0 | 0.0 |
| *Diplopeltoides sp3* | 0.0 | 0.9 | 0.0 |  | 0.0 | 0.0 | 0.0 |  | 0.0 | 0.0 | 0.0 |
| *Diplopeltoides sp7* | 0.0 | 0.0 | 0.8 |  | 0.4 | 0.0 | 0.0 |  | 0.0 | 0.0 | 0.0 |
| *Diplopeltula sp1* | 0.0 | 0.5 | 0.4 |  | 0.0 | 0.0 | 0.8 |  | 0.4 | 0.9 | 0.7 |
| *Diplopeltula sp5* | 0.0 | 0.0 | 0.0 |  | 0.0 | 0.0 | 0.0 |  | 0.0 | 0.5 | 0.0 |
| *Disconema sp1* | 0.0 | 0.0 | 0.0 |  | 0.0 | 1.3 | 0.0 |  | 0.4 | 0.5 | 0.7 |
| *Disconema sp2* | 0.0 | 0.0 | 0.4 |  | 0.8 | 0.0 | 1.1 |  | 0.0 | 0.0 | 0.0 |
| *Disconema sp4* | 0.0 | 0.0 | 0.0 |  | 0.0 | 0.0 | 0.4 |  | 0.0 | 0.0 | 0.0 |
| *Disconema sp5* | 0.0 | 0.0 | 0.0 |  | 0.0 | 0.0 | 0.0 |  | 0.4 | 0.0 | 0.0 |
| *Dorylaimopsis sp1* | 0.4 | 0.9 | 0.0 |  | 0.0 | 0.0 | 0.0 |  | 0.0 | 0.0 | 0.0 |
| *Elzalia sp1* | 1.3 | 0.9 | 3.6 |  | 4.3 | 8.0 | 6.8 |  | 1.9 | 2.7 | 0.0 |
| *Elzalia sp2* | 0.0 | 0.0 | 0.4 |  | 0.4 | 0.0 | 0.0 |  | 0.0 | 0.5 | 0.0 |
| *Elzalia sp3* | 0.0 | 0.0 | 0.4 |  | 0.0 | 0.0 | 0.0 |  | 0.0 | 0.0 | 0.0 |
| *Elzalia sp4* | 0.0 | 0.0 | 0.0 |  | 0.8 | 0.0 | 0.0 |  | 0.0 | 0.5 | 1.4 |
| *Elzalia sp5* | 0.0 | 0.0 | 0.0 |  | 0.0 | 0.0 | 0.0 |  | 0.0 | 0.5 | 0.0 |
| *Elzalia sp7* | 0.0 | 0.0 | 0.0 |  | 0.4 | 0.0 | 0.0 |  | 0.0 | 0.0 | 0.0 |
| *Elzalia sp8* | 0.0 | 0.0 | 1.6 |  | 1.2 | 0.0 | 1.1 |  | 0.4 | 0.5 | 0.0 |
| *Elzalia sp9* | 0.0 | 0.0 | 0.0 |  | 0.0 | 0.0 | 0.0 |  | 0.4 | 0.5 | 1.4 |
| *Enoploides sp1* | 0.4 | 0.0 | 0.4 |  | 0.8 | 0.0 | 0.0 |  | 0.0 | 0.0 | 0.0 |
| *Enoplolaimus sp1* | 0.0 | 0.0 | 0.0 |  | 0.0 | 2.7 | 0.4 |  | 0.0 | 0.9 | 0.0 |
| *Eumorpholaimus sp2* | 0.0 | 0.0 | 0.4 |  | 0.0 | 0.0 | 0.0 |  | 0.0 | 0.0 | 0.0 |
| *Gnomoxyala sp1* | 0.0 | 0.5 | 0.0 |  | 0.0 | 0.0 | 0.0 |  | 0.0 | 0.0 | 0.0 |
| *Graphonema sp1* | 0.4 | 0.0 | 0.0 |  | 1.9 | 0.0 | 4.1 |  | 0.8 | 8.2 | 1.4 |
| *Greeffiella sp1* | 1.7 | 0.9 | 0.0 |  | 0.0 | 0.0 | 0.0 |  | 0.0 | 0.0 | 0.7 |
| *Greeffiellopsis sp1* | 0.4 | 0.0 | 0.0 |  | 0.0 | 0.0 | 0.0 |  | 0.0 | 0.0 | 0.0 |
| *Halalaimus filicaudatus* | 2.2 | 0.0 | 3.6 |  | 5.8 | 2.7 | 3.8 |  | 6.1 | 6.8 | 6.8 |
| *Halalaimus longicaudatus* | 0.0 | 1.9 | 0.4 |  | 0.8 | 2.7 | 0.0 |  | 1.9 | 0.9 | 0.7 |
| *Halalaimus sp1* | 1.3 | 2.4 | 2.0 |  | 0.8 | 2.7 | 1.1 |  | 0.0 | 1.8 | 0.0 |
| *Halalaimus sp4* | 1.3 | 0.0 | 0.0 |  | 0.0 | 0.0 | 0.0 |  | 0.0 | 0.0 | 0.0 |
| *Halalaimus sp9* | 0.9 | 0.0 | 0.0 |  | 0.0 | 0.0 | 0.0 |  | 0.0 | 0.0 | 0.0 |
| *Halichoanolaimus sp1* | 0.0 | 1.4 | 0.8 |  | 0.8 | 0.0 | 0.0 |  | 0.0 | 0.9 | 1.4 |
| *Halichoanolaimus sp2* | 0.0 | 0.0 | 0.0 |  | 0.0 | 0.0 | 0.0 |  | 0.0 | 0.0 | 0.7 |
| *Halichoanolaimus sp5* | 0.0 | 0.0 | 0.4 |  | 0.8 | 0.0 | 1.9 |  | 1.1 | 1.4 | 0.7 |
| *Hopperia sp1* | 0.4 | 4.7 | 1.2 |  | 0.8 | 2.7 | 1.1 |  | 1.1 | 1.8 | 0.0 |
| *Hopperia sp5* | 0.0 | 0.5 | 1.2 |  | 1.6 | 0.0 | 0.8 |  | 2.7 | 1.4 | 3.4 |
| *Hopperia sp6* | 0.0 | 0.0 | 0.0 |  | 0.4 | 0.0 | 0.0 |  | 0.0 | 0.0 | 0.0 |
| *Hypodontolaimus sp1* | 0.0 | 0.0 | 0.0 |  | 0.0 | 0.0 | 0.0 |  | 0.0 | 0.5 | 0.0 |
| *Innocuonema sp1* | 0.0 | 4.3 | 0.0 |  | 0.0 | 0.0 | 0.0 |  | 0.0 | 0.0 | 0.0 |
| *Leptolaimoides sp1* | 0.4 | 0.0 | 0.0 |  | 0.4 | 0.0 | 0.0 |  | 1.5 | 0.0 | 0.0 |
| *Leptolaimus sp1* | 8.7 | 4.7 | 0.0 |  | 0.4 | 1.3 | 0.4 |  | 0.0 | 0.0 | 0.0 |
| *Leptolaimus sp2* | 0.4 | 0.0 | 0.0 |  | 0.0 | 0.0 | 1.1 |  | 0.0 | 0.0 | 0.0 |
| *Leptolaimus sp3* | 0.0 | 0.0 | 0.0 |  | 0.0 | 0.0 | 0.4 |  | 0.0 | 0.0 | 0.0 |
| *Leptolaimus sp4* | 2.2 | 0.0 | 0.0 |  | 0.0 | 0.0 | 0.0 |  | 0.0 | 0.0 | 0.0 |
| *Leptolaimus sp5* | 0.4 | 1.9 | 0.0 |  | 0.8 | 0.0 | 0.8 |  | 0.0 | 0.0 | 0.0 |
| *Leptolaimus sp7* | 0.0 | 0.0 | 0.4 |  | 0.0 | 0.0 | 0.0 |  | 0.4 | 0.0 | 0.0 |
| *Leptosomatides sp1* | 0.0 | 0.0 | 0.0 |  | 0.0 | 0.0 | 0.0 |  | 0.0 | 0.9 | 1.4 |
| *Linhystera sp1* | 0.0 | 0.0 | 0.4 |  | 0.4 | 0.0 | 0.0 |  | 1.1 | 4.1 | 0.7 |
| *Linhystera sp2* | 0.0 | 0.0 | 0.0 |  | 0.0 | 0.0 | 0.0 |  | 2.3 | 3.2 | 1.4 |
| *Linhystera sp4* | 0.0 | 0.0 | 0.0 |  | 0.0 | 0.0 | 0.0 |  | 0.4 | 0.0 | 0.0 |
| *Litinium sp1* | 0.0 | 0.0 | 0.0 |  | 0.0 | 0.0 | 0.4 |  | 0.0 | 0.0 | 0.0 |
| *Litinium sp7* | 0.4 | 0.0 | 0.0 |  | 0.0 | 0.0 | 0.0 |  | 0.0 | 0.0 | 0.7 |
| *Longicyatholaimus sp1* | 0.0 | 0.0 | 0.0 |  | 0.0 | 0.0 | 0.0 |  | 1.1 | 0.0 | 0.0 |
| *Marylynnia sp1* | 0.0 | 0.5 | 0.0 |  | 0.4 | 0.0 | 3.0 |  | 1.9 | 1.4 | 1.4 |
| *Marylynnia sp2* | 0.0 | 0.0 | 0.0 |  | 0.4 | 0.0 | 0.0 |  | 0.0 | 0.0 | 0.0 |
| *Marylynnia sp5* | 0.0 | 0.0 | 0.0 |  | 0.0 | 0.0 | 0.0 |  | 0.4 | 0.5 | 0.7 |
| *Megadesmolaimus sp1* | 0.0 | 0.5 | 0.0 |  | 0.0 | 0.0 | 0.0 |  | 0.0 | 0.0 | 0.0 |
| *Megadesmolaimus sp2* | 0.0 | 0.0 | 0.0 |  | 0.4 | 0.0 | 0.0 |  | 0.0 | 0.0 | 0.0 |
| *Metachromadora sp1* | 0.0 | 0.0 | 0.0 |  | 0.0 | 0.0 | 0.0 |  | 0.4 | 0.0 | 0.0 |
| *Metacyatholaimus sp1* | 0.4 | 0.0 | 0.0 |  | 0.0 | 0.0 | 0.0 |  | 0.0 | 0.0 | 0.0 |
| *Metadesmolaimus sp1* | 0.0 | 0.0 | 0.8 |  | 0.8 | 0.0 | 0.0 |  | 0.0 | 0.0 | 0.0 |
| *Metadesmolaimus sp3* | 0.0 | 0.0 | 0.0 |  | 0.8 | 0.0 | 0.0 |  | 0.0 | 0.0 | 0.0 |
| *Metalinhomoeus sp1* | 0.4 | 0.0 | 0.4 |  | 0.8 | 0.0 | 0.4 |  | 0.8 | 0.0 | 0.0 |
| *Metasphaerolaimus sp1* | 0.0 | 0.0 | 0.0 |  | 0.0 | 0.0 | 0.0 |  | 1.1 | 0.0 | 2.7 |
| *Metasphaerolaimus sp2* | 0.0 | 0.0 | 0.0 |  | 0.0 | 0.0 | 0.0 |  | 0.0 | 0.0 | 0.7 |
| *Metasphaerolaimus sp5* | 0.0 | 0.0 | 0.0 |  | 0.0 | 0.0 | 0.4 |  | 0.0 | 0.0 | 0.0 |
| *Micoletzkyia sp1* | 0.0 | 0.5 | 0.0 |  | 0.0 | 0.0 | 0.0 |  | 0.0 | 0.0 | 0.0 |
| *Microlaimus sp1* | 0.0 | 0.0 | 5.6 |  | 0.8 | 1.3 | 2.6 |  | 0.0 | 0.9 | 0.7 |
| *Microlaimus sp3* | 0.0 | 0.0 | 1.2 |  | 0.0 | 0.0 | 0.0 |  | 0.0 | 0.0 | 0.0 |
| *Minolaimus sp1* | 0.4 | 0.0 | 0.0 |  | 0.0 | 0.0 | 0.0 |  | 0.0 | 0.0 | 0.0 |
| *Minolaimus sp2* | 0.4 | 0.0 | 0.0 |  | 0.0 | 0.0 | 0.0 |  | 0.0 | 0.0 | 0.0 |
| *Minolaimus sp5* | 0.4 | 0.0 | 0.0 |  | 0.0 | 0.0 | 0.0 |  | 0.0 | 0.0 | 0.0 |
| *Molgolaimus sp1* | 2.2 | 0.0 | 0.0 |  | 0.0 | 0.0 | 0.0 |  | 0.0 | 0.0 | 0.0 |
| *Molgolaimus sp8* | 0.4 | 0.0 | 0.0 |  | 0.0 | 0.0 | 0.0 |  | 0.0 | 0.0 | 0.0 |
| *Monhystera sp1* | 0.4 | 0.5 | 0.0 |  | 0.0 | 0.0 | 0.0 |  | 0.0 | 0.0 | 0.0 |
| *Nemanema sp1* | 0.0 | 0.0 | 0.0 |  | 0.0 | 0.0 | 0.0 |  | 0.0 | 0.0 | 2.1 |
| *Oncholaimellus sp1* | 0.0 | 0.0 | 0.0 |  | 0.0 | 0.0 | 0.0 |  | 0.0 | 0.0 | 0.7 |
| *Oncholaimellus sp2* | 0.0 | 0.5 | 0.0 |  | 0.0 | 1.3 | 0.0 |  | 0.0 | 0.0 | 0.0 |
| *Oncholaimus sp1* | 0.4 | 0.0 | 0.0 |  | 0.0 | 0.0 | 0.0 |  | 0.0 | 0.0 | 0.0 |
| *Oxystomina sp1* | 0.4 | 0.5 | 0.4 |  | 1.6 | 2.7 | 0.4 |  | 0.4 | 0.9 | 0.7 |
| *Oxystomina sp2* | 0.0 | 1.9 | 0.0 |  | 0.0 | 0.0 | 1.1 |  | 0.0 | 0.0 | 0.0 |
| *Oxystomina sp4* | 0.0 | 0.5 | 1.6 |  | 0.0 | 0.0 | 0.8 |  | 0.0 | 0.0 | 0.0 |
| *Oxystomina sp5* | 0.0 | 0.5 | 0.0 |  | 0.0 | 0.0 | 0.4 |  | 0.0 | 0.0 | 0.0 |
| *Oxystomina sp6* | 0.0 | 0.0 | 0.4 |  | 0.0 | 0.0 | 0.0 |  | 0.0 | 0.0 | 0.0 |
| *Pandolaimus sp1* | 0.0 | 0.0 | 0.0 |  | 0.0 | 0.0 | 0.0 |  | 0.4 | 0.0 | 0.0 |
| *Paracanthonchus sp1* | 0.0 | 0.0 | 0.0 |  | 1.2 | 1.3 | 0.4 |  | 0.0 | 0.5 | 0.0 |
| *Paracyatholaimus sp1* | 0.9 | 0.0 | 0.8 |  | 0.0 | 0.0 | 0.0 |  | 0.0 | 1.4 | 0.0 |
| *Paracyatholaimus sp3* | 0.0 | 0.0 | 0.4 |  | 0.0 | 0.0 | 0.0 |  | 0.0 | 0.0 | 0.0 |
| *Paralimononheus sp1* | 0.0 | 0.0 | 0.0 |  | 0.0 | 0.0 | 0.0 |  | 0.4 | 0.0 | 0.0 |
| *Paralongicyatholaimus sp1* | 0.0 | 1.9 | 0.0 |  | 1.6 | 0.0 | 0.0 |  | 0.4 | 0.9 | 0.0 |
| *Paralongicyatholaimus sp5* | 0.0 | 0.0 | 0.0 |  | 0.4 | 0.0 | 0.0 |  | 0.0 | 0.0 | 0.0 |
| *Paramonohystera sp1* | 0.0 | 0.0 | 0.0 |  | 0.0 | 0.0 | 0.0 |  | 1.1 | 0.9 | 0.0 |
| *Paramonohystera sp4* | 0.0 | 0.0 | 0.8 |  | 0.0 | 0.0 | 0.0 |  | 0.0 | 0.0 | 0.0 |
| *Pareudesmoscolex sp1* | 3.9 | 0.0 | 0.0 |  | 0.0 | 0.0 | 0.0 |  | 0.0 | 0.0 | 0.0 |
| *Parodontophora sp1* | 0.9 | 0.0 | 0.0 |  | 0.0 | 0.0 | 0.0 |  | 0.8 | 0.5 | 0.0 |
| *Parodontophora sp3* | 0.0 | 0.0 | 0.0 |  | 0.0 | 0.0 | 0.0 |  | 0.4 | 0.0 | 0.0 |
| *Pierrickia sp1* | 4.3 | 2.8 | 4.4 |  | 3.9 | 0.0 | 5.3 |  | 3.8 | 0.5 | 1.4 |
| *Pierrickia sp2* | 0.4 | 0.9 | 0.0 |  | 0.0 | 1.3 | 0.4 |  | 0.8 | 0.0 | 0.0 |
| *Pierrickia sp3* | 0.0 | 1.9 | 2.0 |  | 1.9 | 0.0 | 1.5 |  | 3.4 | 3.7 | 1.4 |
| *Pierrickia sp4* | 3.5 | 2.8 | 0.4 |  | 1.9 | 1.3 | 0.4 |  | 0.4 | 0.5 | 2.1 |
| *Pierrickia sp5* | 0.4 | 1.4 | 1.2 |  | 0.0 | 0.0 | 0.4 |  | 1.1 | 0.0 | 0.0 |
| *Pierrickia sp8* | 0.0 | 0.0 | 0.0 |  | 0.0 | 0.0 | 0.0 |  | 0.4 | 0.0 | 0.0 |
| *Platycoma sp1* | 0.0 | 0.0 | 0.0 |  | 0.0 | 0.0 | 0.4 |  | 0.0 | 0.0 | 0.0 |
| *Polysigma sp1* | 0.4 | 0.0 | 0.0 |  | 0.0 | 0.0 | 0.0 |  | 0.0 | 0.0 | 0.0 |
| *Polysigma sp2* | 0.4 | 0.0 | 0.0 |  | 0.0 | 0.0 | 0.0 |  | 0.0 | 0.0 | 0.0 |
| *Pselionema sp1* | 2.2 | 2.4 | 0.0 |  | 0.0 | 0.0 | 0.0 |  | 0.0 | 0.0 | 0.7 |
| *Pselionema sp3* | 0.0 | 0.0 | 0.0 |  | 1.9 | 0.0 | 1.1 |  | 1.9 | 0.9 | 0.7 |
| *Pselionema sp5* | 0.0 | 0.0 | 0.0 |  | 0.4 | 0.0 | 0.0 |  | 0.4 | 0.0 | 0.0 |
| *Quadricoma sp1* | 0.0 | 0.0 | 0.0 |  | 0.4 | 0.0 | 0.0 |  | 0.0 | 0.0 | 0.0 |
| *Rhabditis sp1* | 0.0 | 0.5 | 0.4 |  | 2.3 | 2.7 | 3.8 |  | 2.7 | 2.7 | 0.7 |
| *Rhabdodemania sp1* | 0.4 | 0.0 | 0.0 |  | 0.4 | 1.3 | 0.0 |  | 0.0 | 0.0 | 2.1 |
| *Rhabdodemania sp2* | 0.4 | 0.0 | 0.4 |  | 0.0 | 0.0 | 0.0 |  | 0.0 | 0.0 | 0.0 |
| *Rhabdodemania sp3* | 2.2 | 0.9 | 0.0 |  | 0.0 | 0.0 | 0.0 |  | 0.0 | 0.0 | 0.0 |
| *Rhips sp1* | 0.4 | 0.0 | 1.2 |  | 0.0 | 0.0 | 0.0 |  | 0.0 | 0.5 | 0.7 |
| *Rhynchonema sp1* | 0.4 | 0.0 | 0.0 |  | 0.0 | 0.0 | 0.0 |  | 0.0 | 0.0 | 0.0 |
| *Richtersia sp1* | 8.7 | 5.7 | 0.4 |  | 0.0 | 0.0 | 0.0 |  | 0.0 | 0.5 | 0.0 |
| *Richtersia sp3* | 0.0 | 0.5 | 0.0 |  | 0.0 | 0.0 | 0.0 |  | 0.0 | 0.0 | 0.0 |
| *Richtersia sp4* | 0.0 | 0.0 | 0.0 |  | 0.0 | 2.7 | 0.4 |  | 0.8 | 0.0 | 0.7 |
| *Richtersia sp5* | 0.0 | 2.4 | 1.6 |  | 0.4 | 0.0 | 3.4 |  | 3.1 | 0.0 | 0.7 |
| *Richtersia sp6* | 0.0 | 0.9 | 0.0 |  | 1.2 | 0.0 | 1.1 |  | 2.7 | 0.9 | 0.7 |
| *Richtersia sp7* | 0.0 | 0.0 | 0.0 |  | 0.0 | 0.0 | 0.0 |  | 0.4 | 0.0 | 0.0 |
| *Richtersia sp8* | 0.0 | 1.9 | 4.0 |  | 4.7 | 6.7 | 5.6 |  | 5.0 | 1.4 | 2.7 |
| *Sabatieria sp1* | 5.2 | 5.7 | 3.2 |  | 3.9 | 6.7 | 0.8 |  | 1.5 | 4.1 | 6.2 |
| *Sabatieria sp2* | 0.4 | 0.9 | 0.0 |  | 0.0 | 0.0 | 0.0 |  | 0.0 | 0.0 | 2.1 |
| *Sabatieria sp3* | 1.7 | 0.0 | 0.0 |  | 0.0 | 0.0 | 0.0 |  | 0.4 | 0.5 | 0.0 |
| *Sabatiera sp 4* | 0.0 | 0.5 | 0.4 |  | 0.0 | 0.0 | 0.0 |  | 0.8 | 0.0 | 0.0 |
| *Sabatiera sp 5* | 0.0 | 0.0 | 0.4 |  | 0.0 | 0.0 | 0.0 |  | 0.0 | 0.0 | 0.0 |
| *Sabatiera sp 6* | 0.0 | 0.0 | 2.4 |  | 0.0 | 0.0 | 0.0 |  | 1.9 | 1.4 | 0.7 |
| *Setoplectus sp1* | 0.9 | 0.0 | 0.0 |  | 0.0 | 0.0 | 0.0 |  | 0.0 | 0.0 | 0.0 |
| *Setosabatieria sp1* | 2.2 | 2.4 | 1.6 |  | 3.1 | 5.3 | 0.8 |  | 1.9 | 1.8 | 0.0 |
| *Setosabatieria sp2* | 0.0 | 0.0 | 0.0 |  | 0.0 | 0.0 | 0.0 |  | 0.4 | 0.0 | 0.0 |
| *Setosabatieria sp4* | 0.0 | 0.0 | 0.0 |  | 0.0 | 0.0 | 0.0 |  | 0.0 | 0.5 | 0.0 |
| *Setosabatieria sp5* | 0.0 | 0.0 | 5.6 |  | 2.7 | 2.7 | 2.3 |  | 1.5 | 0.5 | 0.0 |
| *Setosabatieria sp6* | 0.0 | 0.0 | 0.0 |  | 0.0 | 0.0 | 0.0 |  | 0.0 | 0.5 | 0.0 |
| *Sphaerolaimus sp1* | 1.3 | 1.9 | 3.6 |  | 0.4 | 4.0 | 2.3 |  | 1.5 | 2.3 | 0.0 |
| *Sphaerolaimus sp3* | 0.0 | 0.0 | 0.4 |  | 0.0 | 0.0 | 0.0 |  | 0.4 | 0.9 | 1.4 |
| *Sphaerolaimus sp4* | 0.0 | 0.0 | 0.4 |  | 0.0 | 0.0 | 0.0 |  | 0.0 | 0.0 | 0.0 |
| *Sphaerolaimus sp8* | 0.0 | 0.0 | 0.0 |  | 0.0 | 0.0 | 0.0 |  | 0.8 | 1.4 | 0.7 |
| *Spilophorella sp1* | 0.0 | 0.9 | 3.6 |  | 1.6 | 0.0 | 1.9 |  | 1.1 | 0.9 | 1.4 |
| *Steineira sp1* | 0.0 | 0.0 | 0.0 |  | 0.0 | 0.0 | 0.0 |  | 0.4 | 0.0 | 0.0 |
| *Steineira sp2* | 0.0 | 0.0 | 0.0 |  | 0.0 | 0.0 | 0.0 |  | 0.0 | 0.5 | 0.0 |
| *Stephanolaimus sp1* | 0.0 | 0.5 | 0.0 |  | 0.0 | 0.0 | 0.0 |  | 0.0 | 1.4 | 0.7 |
| *Synonchiella sp1* | 0.0 | 0.9 | 0.0 |  | 0.0 | 0.0 | 0.0 |  | 0.0 | 0.0 | 0.0 |
| *Syringolaimus sp1* | 0.4 | 0.5 | 0.0 |  | 0.0 | 0.0 | 0.0 |  | 0.0 | 0.0 | 0.0 |
| *Terschellingia sp1* | 0.4 | 0.5 | 0.4 |  | 0.0 | 0.0 | 0.0 |  | 0.4 | 0.0 | 0.0 |
| *Terschellingia sp2* | 0.0 | 0.0 | 0.4 |  | 0.8 | 0.0 | 0.0 |  | 0.0 | 0.0 | 0.0 |
| *Terschellingia sp4* | 0.4 | 2.4 | 0.4 |  | 0.0 | 5.3 | 2.3 |  | 3.1 | 0.9 | 0.0 |
| *Thalassironus sp1* | 0.0 | 0.5 | 0.0 |  | 0.0 | 0.0 | 0.0 |  | 0.0 | 0.0 | 0.0 |
| *Theristus sp1* | 0.0 | 0.0 | 0.0 |  | 0.4 | 0.0 | 0.8 |  | 0.0 | 1.4 | 2.1 |
| *Theristus sp2* | 0.0 | 0.0 | 0.0 |  | 1.6 | 1.3 | 2.6 |  | 1.1 | 1.4 | 0.7 |
| *Theristus sp8* | 0.0 | 0.0 | 0.0 |  | 0.0 | 0.0 | 0.4 |  | 0.0 | 0.0 | 0.0 |
| *Tricoma sp1* | 2.2 | 0.5 | 0.0 |  | 0.8 | 1.3 | 0.0 |  | 0.8 | 0.5 | 0.7 |
| *Trissonchulus sp1* | 0.0 | 0.0 | 0.0 |  | 0.0 | 0.0 | 0.8 |  | 0.0 | 0.0 | 0.0 |
| *Vasostoma sp1* | 1.3 | 0.0 | 0.4 |  | 0.0 | 0.0 | 0.0 |  | 0.0 | 0.0 | 0.0 |
| *Wieseria sp1* | 0.9 | 0.5 | 0.0 |  | 0.0 | 0.0 | 0.0 |  | 0.0 | 0.0 | 0.0 |
