# Supplementary material for: High Meiofaunal and Nematodes Diversity around Mesophotic Coral Oases in the Mediterranean Sea
Source: PLoS One. 2013 Jun 18;8(6):e66553. doi: 10.1371/journal.pone.0066553 (PMC3688901; doi:10.1371/journal.pone.0066553)
Supplement: Table S3 — Output of two-way ANOVAs carried out on meiofaunal abundance and biomass. df = degree of freedom, MS = mean square, F = ANOVA F statistic, P = probability level. * = P<0.05, ns = not significant. (DOCX) [file pone.0066553.s003.docx]

**Appendix S3**

**Table S3** Output of two-way ANOVAs carried out on meiofaunal abundance and biomass. df = degree of freedom, MS = mean square, F = ANOVA F statistic, P = probability level. * = P <0.05, ns = not significant.

|  |  | Main test | | | |  |  | Pairwise comparisons | | |
| --- | --- | --- | --- | --- | --- | --- | --- | --- | --- | --- |
|  |  |  |  |  |  |  |  | Transect 1 | Transect 2 | Transect 3 |
|  | Source | df | MS | F | P |  | Pair wise | P | P | P |
| Abundance | Transect | 2 | 7309 | 0.132 | ns |  | 1, 100 | ns | ns | ns |
|  | Distance | 2 | 64535 | 0.663 | ns |  | 1, 200 | ns | ns | ns |
|  | Transect x Distance | 4 | 97274 | 1.762 | ns |  | 100, 200 | ns | ns | ns |
|  | Residual | 18 | 55221 |  |  |  |  |  |  |  |
|  | Total | 26 |  |  |  |  |  |  |  |  |
| Biomass | Transect | 2 | 1088 | 0.71 | ns |  | 1, 100 | ns | * | ns |
|  | Distance | 2 | 115 | 0.03 | ns |  | 1, 200 | ns | * | ns |
|  | Transect x Distance | 4 | 3757 | 2.453 | * |  | 100, 200 | ns | ns | ns |
|  | Residual | 18 | 1532 |  |  |  |  |  |  |  |
|  | Total | 26 |  |  |  |  |  |  |  |  |
