# Supplementary material for: High Meiofaunal and Nematodes Diversity around Mesophotic Coral Oases in the Mediterranean Sea
Source: PLoS One. 2013 Jun 18;8(6):e66553. doi: 10.1371/journal.pone.0066553 (PMC3688901; doi:10.1371/journal.pone.0066553)
Supplement: Table S4 — Output of two-way ANOVAs carried out on richness of meiofaunal taxa and nematode diversity indexes. df = degree of freedom, MS = mean square, F = ANOVA F statistic, P = probability level. * = P<0.05, ns = not significant. SR = species richness, H = Shannon-Wiener index, J = Pielou's index, ITD = index of trophic diversity, MI = maturity index. (DOCX) [file pone.0066553.s004.docx]

**Appendix S4**

**Table S4** Output of two-way ANOVAs carried out on richness of meiofaunal taxa and nematode diversity indexes. df = degree of freedom, MS = mean square, F = ANOVA F statistic, P = probability level. * = P <0.05, ns = not significant. SR = species richness, H = Shannon-Wiener index, J = Pielou’s index, ITD = index of trophic diversity, MI = maturity index.

|  |  |  |  |  |  |  | **Pair wise** | |  | **Pair wise Transect x Distance** | | |
| --- | --- | --- | --- | --- | --- | --- | --- | --- | --- | --- | --- | --- |
|  | **Source** | **DF** | **MS** | **F** | **P** |  | **Transect** | **Distance** |  | **Transect 1** | **Transect 2** | **Transect 3** |
| **Richness of taxa** | **Transect (T)** | 2 | 8.93 | 3.35 | * |  | 3>2 |  |  |  |  |  |
|  | **Distance (D)** | 2 | 1.15 | 0.43 | ns |  |  | ns |  |  |  |  |
|  | **T X D** | 4 | 10.4 | 3.89 | * |  |  |  |  | ns | 1>100,200 | ns |
|  | **Residual** | 18 | 2.67 |  |  |  |  |  |  |  |  |  |
|  | **Total** | 26 |  |  |  |  |  |  |  |  |  |  |
| **SR** | **Transect (T)** | 2 | 50 | 0.53 | ns |  | ns |  |  |  |  |  |
|  | **Distance (D)** | 2 | 362 | 3.85 | * |  |  | 1>100 |  |  |  |  |
|  | **T X D** | 4 | 408 | 4.34 | * |  |  |  |  | ns | 1,200>100 | ns |
|  | **Residual** | 18 | 94 |  |  |  |  |  |  |  |  |  |
|  | **Total** | 26 |  |  |  |  |  |  |  |  |  |  |
| **ES 100** | **Transect (T)** | 2 | 50 | 0.53 | ns |  | ns |  |  |  |  |  |
|  | **Distance (D)** | 2 | 362 | 3.85 | * |  |  | 1>100 |  |  |  |  |
|  | **T X D** | 4 | 408 | 4.34 | * |  |  |  |  | ns | 1,200>100 | ns |
|  | **Residual** | 18 | 94 |  |  |  |  |  |  |  |  |  |
|  | **Total** | 26 |  |  |  |  |  |  |  |  |  |  |
| **H** | **Transect (T)** | 2 | 6.64 | 0.96 | ns |  | ns |  |  |  |  |  |
|  | **Distance (D)** | 2 | 25.4 | 3.68 | * |  |  | 1>100 |  |  |  |  |
|  | **T X D** | 4 | 30.7 | 4.44 | * |  |  |  |  | ns | 1,200>100 | ns |
|  | **Residual** | 18 | 6.9 |  |  |  |  |  |  |  |  |  |
|  | **Total** | 26 |  |  |  |  |  |  |  |  |  |  |
| **J** | **Transect (T)** | 2 | 0.14 | 4.5 | * |  | 1,3>2 |  |  |  |  |  |
|  | **Distance (D)** | 2 | 0.12 | 3.77 | * |  |  | 1>100 |  |  |  |  |
|  | **T X D** | 4 | 0.13 | 4.13 | * |  |  |  |  | ns | 1,200>100 | ns |
|  | **Residual** | 18 | 0.03 |  |  |  |  |  |  |  |  |  |
|  | **Total** | 26 |  |  |  |  |  |  |  |  |  |  |
| **1 - ITD** | **Transect (T)** | 2 | 0.07 | 4.26 | * |  | 1,3>2 |  |  |  |  |  |
|  | **Distance (D)** | 2 | 0.07 | 4.4 | * |  |  | 1,200>100 |  |  |  |  |
|  | **T X D** | 4 | 0.08 | 4.78 | ** |  |  |  |  | ns | 1,200>100 | ns |
|  | **Residual** | 18 | 0.02 |  |  |  |  |  |  |  |  |  |
|  | **Total** | 26 |  |  |  |  |  |  |  |  |  |  |
| **MI** | **Transect (T)** | 2 | 0.28 | 4.31 | * |  | 1,3>2 |  |  |  |  |  |
|  | **Distance (D)** | 2 | 0.26 | 3.97 | * |  |  | 200>100 |  |  |  |  |
|  | **T X D** | 4 | 0.26 | 3.98 | * |  |  |  |  | ns | 1,200>100 | ns |
|  | **Residual** | 18 | 0.07 |  |  |  |  |  |  |  |  |  |
|  | **Total** | 26 |  |  |  |  |  |  |  |  |  |  |
